# Strategies of pretreatment of feedstocks for optimized bioethanol production: distinct and integrated approaches

**DOI:** 10.1186/s13068-023-02295-2

**Published:** 2023-03-13

**Authors:** Akanksha Shukla, Deepak Kumar, Madhuri Girdhar, Anil Kumar, Abhineet Goyal, Tabarak Malik, Anand Mohan

**Affiliations:** 1 School of Bioengineering and Biosciences, Lovely Professional University, Phagwara, 144411 India; 2 School of Chemical Engineering and Physical Sciences, Lovely Professional University, Phagwara, 144411 India; 3 Gene Regulation Laboratory, National Institute of Immunology, New Delhi, 110067 India; 4 SAGE School of Science, SAGE University Bhopal, Sahara Bypass Road Katara Hills, Extension, Bhopal, Madhya Pradesh 462022 India; 5 Department of Biomedical Sciences, Institute of Health, Jimma University, Jimma, Ethiopia

**Keywords:** Lignocellulosic structure, Physical pretreatment, Chemical pretreatment, Biological pretreatment, Bioethanol, Biofuels

## Abstract

Bioethanol is recognized as a valuable substitute for renewable energy sources to meet the fuel and energy demand of the nation, considered an environmentally friendly resource obtained from agricultural residues such as sugarcane bagasse, rice straw, husk, wheat straw and corn stover. The energy demand is sustained using lignocellulosic biomass to produce bioethanol. Lignocellulosic biomass (LCBs) is the point of attention in replacing the dependence on fossil fuels. The recalcitrant structure of the lignocellulosic biomass is disrupted using effective pretreatment techniques that separate complex interlinked structures among cellulose, hemicellulose, and lignin. Pretreatment of biomass involves various physical, chemical, biological, and physiochemical protocols which are of importance, dependent upon their individual or combined dissolution effect. Physical pretreatment involves a reduction in the size of the biomass using mechanical, extrusion, irradiation, and sonification methods while chemical pretreatment involves the breaking of various bonds present in the LCB structure. This can be obtained by using an acidic, alkaline, ionic liquid, and organosolvent methods. Biological pretreatment is considered an environment-friendly and safe process involving various bacterial and fungal microorganisms. Distinct pretreatment methods, when combined and utilized in synchronization lead to more effective disruption of LCB, making biomass more accessible for further processing. These could be utilized in terms of their effectiveness for a particular type of cellulosic fiber and are namely steam explosion, liquid hot water, ammonia fibre explosion, CO_2_ explosion, and wet air oxidation methods. The present review encircles various distinct and integrated pretreatment processes developed till now and their advancement according to the current trend and future aspects to make lignocellulosic biomass available for further hydrolysis and fermentation.

**Supplementary Information:**

The online version contains supplementary material available at 10.1186/s13068-023-02295-2.

## Introduction

**Fig. 1:**
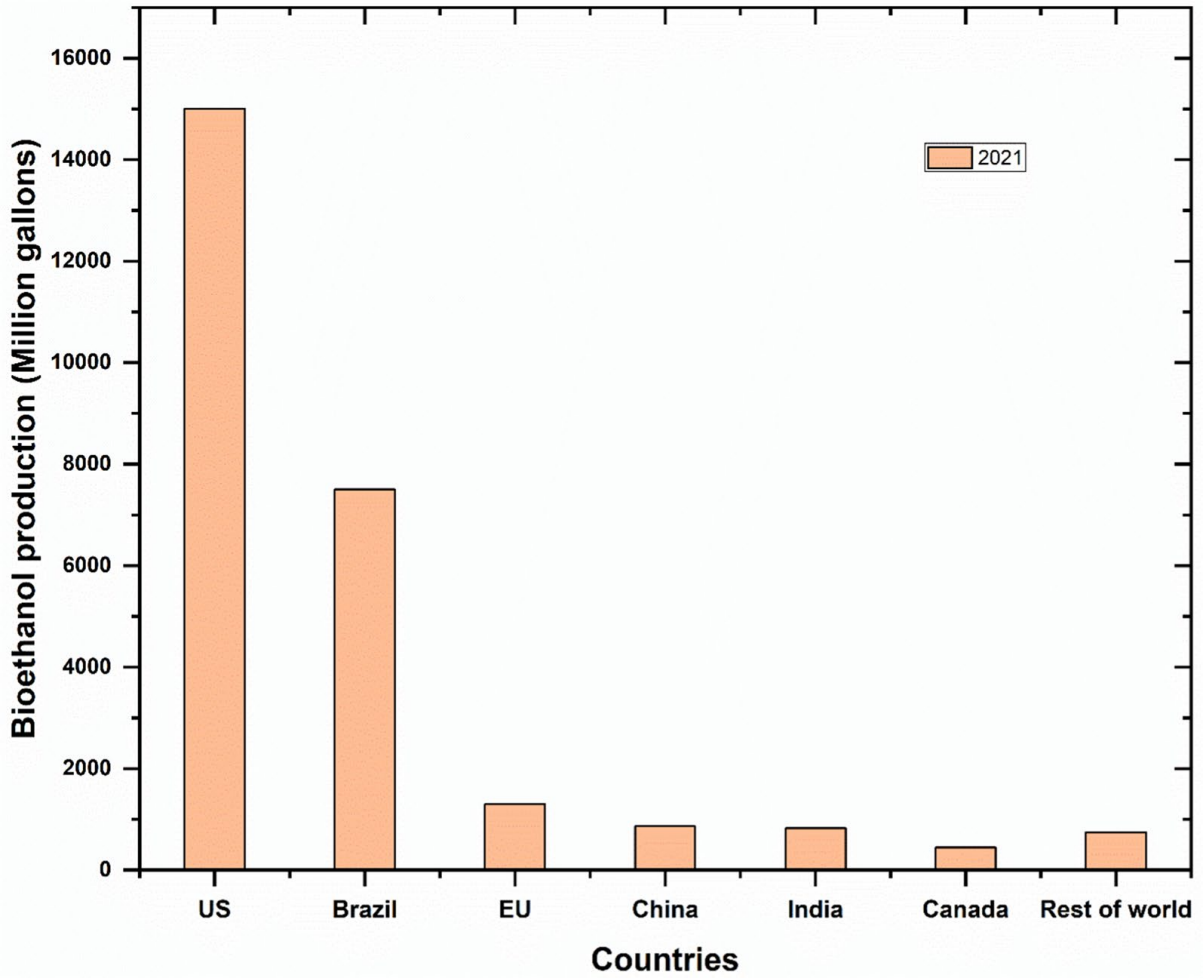
Illustration of global biofuel production relative to different countries in the year 2021

**Fig. 2:**
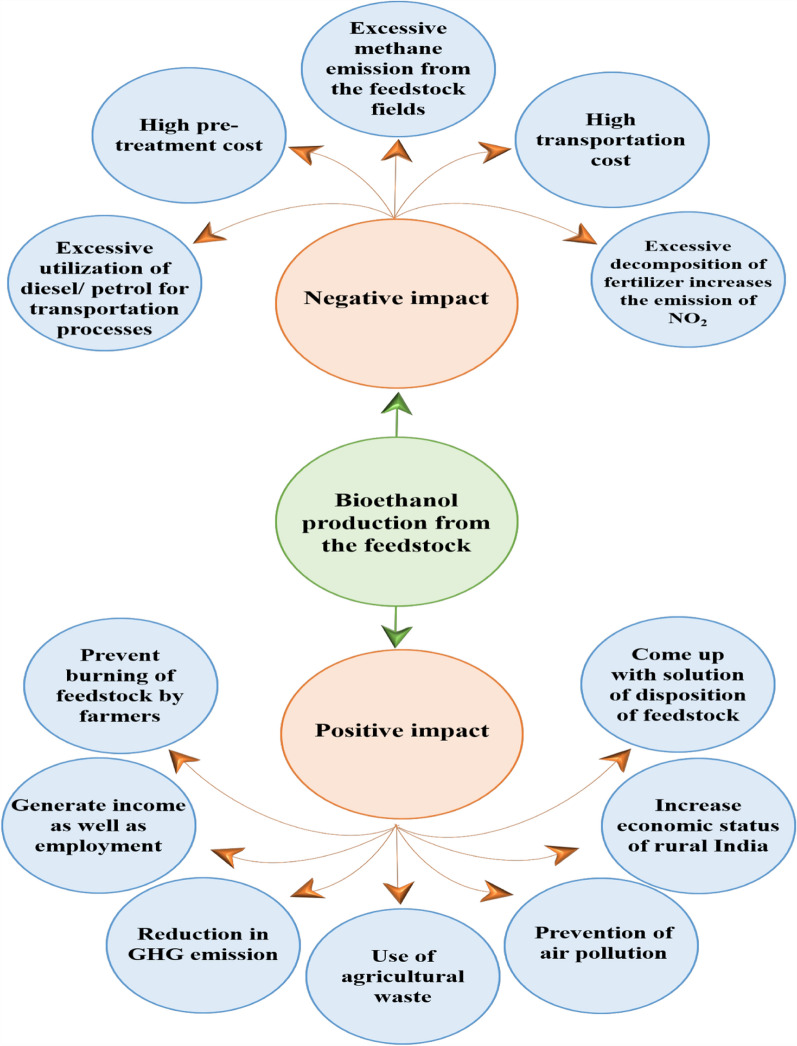
Illustration of positive and negative impact of bioethanol production from feedstock

Agricultural waste is one of the most abundant lignocellulosic biomasses available in India and an attractive alternative to renewable energy generation. Renewable energy generated from agricultural biomasses has the possibility of substituting fossil fuel generation [1]. But due to a lack of awareness, these are burned and dumped in the open environment leading to high greenhouse gas (GHG) emissions. Many countries have planned to reduce GHG emissions by switching to cleantech sources, i.e., ethanol. Ethanol is produced from lignocellulosic waste, the most abandoned renewable biomass, derived from agricultural feedstock such as wheat husk, rice straw, sugarcane bagasse, and corn stover. This organic waste is derived from biological sources, primarily plant biomass, being the most readily available global source of renewable materials, with an estimated annual worldwide production of 1010 MT. On evaluating the total production of various agricultural residues globally, the sugarcane bagasse (SCB) biomass is considered one of the abundant agricultural residues that hold the key to solving the global energy problem and environmental concern [2]. According to recent research, the potential for lignocellulose biomass contributed from SCB worldwide annually is 243 million tonnes, which translates to producing 4.3 EJ of energy, which covers 6.8% of the present global supply of bioenergy [3]. Similarly, corn stover production in 2021–22 was approximately 120 MT globally and has the potential to produce 23–53 billion tons of bioethanol in the United States alone [4]. Approximately 512.8 MT of rice is produced globally every year [5] and according to IRRI, the typical rice grain to straw production ratio is 0.7:1.4 [6]. Thus, it is estimated to produce 1025.6 MT of straw that is burned by the local farmers which if utilized rationally could add up to the global bioethanol production [7]. Among these agricultural waste obtained from food crops, peanut shell is considered bulky waste, producing about 230–300 gm/kg of peanuts with 50.34 MMT of peanut produced worldwide in the 2021–2022 season which can also add up to the cause [8]. It could be easily comprehended that utilizing agricultural residue for ethanol production could be one of the most promising sustainable energy processes due to unending supplies of available lignocellulosic biomass wastes. Global biofuel production relative to different countries in the year 2021 is illustrated in Fig. 1, taking reference from Statista report on world biofuel production by various countries [9] and bioethanol production influence with both positive and negative impact is illustrated in Fig. 2.

Lignocellulosic biomass has complex biochemical and highly heterogeneous structures, characterized by using both chemical and physical structural properties. The structural properties of biomass which include chemical composition, fiber characterization, and cell proposition show a significant effect on the further saccharification process [10]. It is concluded that feedstock with higher cellulose, hemicellulose, lower lignin, and silica content is suitable for bioethanol production. It is estimated that the biochemical structure with the compositional analysis of cellulose (32–47%), hemicellulose (19–27%), and lignin (5–24%) are suitable feedstock for bioethanol production. Since most agricultural wastes contain ≥ 50% fermentable sugars but due to their recalcitrant structure, it is not used further for any chemical and biological process to ferment sugar [11]. However, to make it suitable for further process of hydrolysis and fermentation, a prerequisite step, i.e., pretreatment is required. An ideal pretreatment step dwindles the connective link between lignocellulosic recalcitrant structure and makes feedstock available for further process, i.e., enzymatic accessibility and saccharification process with less inhibitor formation and increase in the recovery rate of cellulose and hemicellulose [12]. The process can be cost-effective by deploying advanced techniques of pretreatment. According to various reports, effective pretreatment reduces the size of the biomass, minimizes sugar loss, and maximizes lignin removal along with a reduction in the formation of inhibitors, thereby making the process economical.

**Fig. 3:**
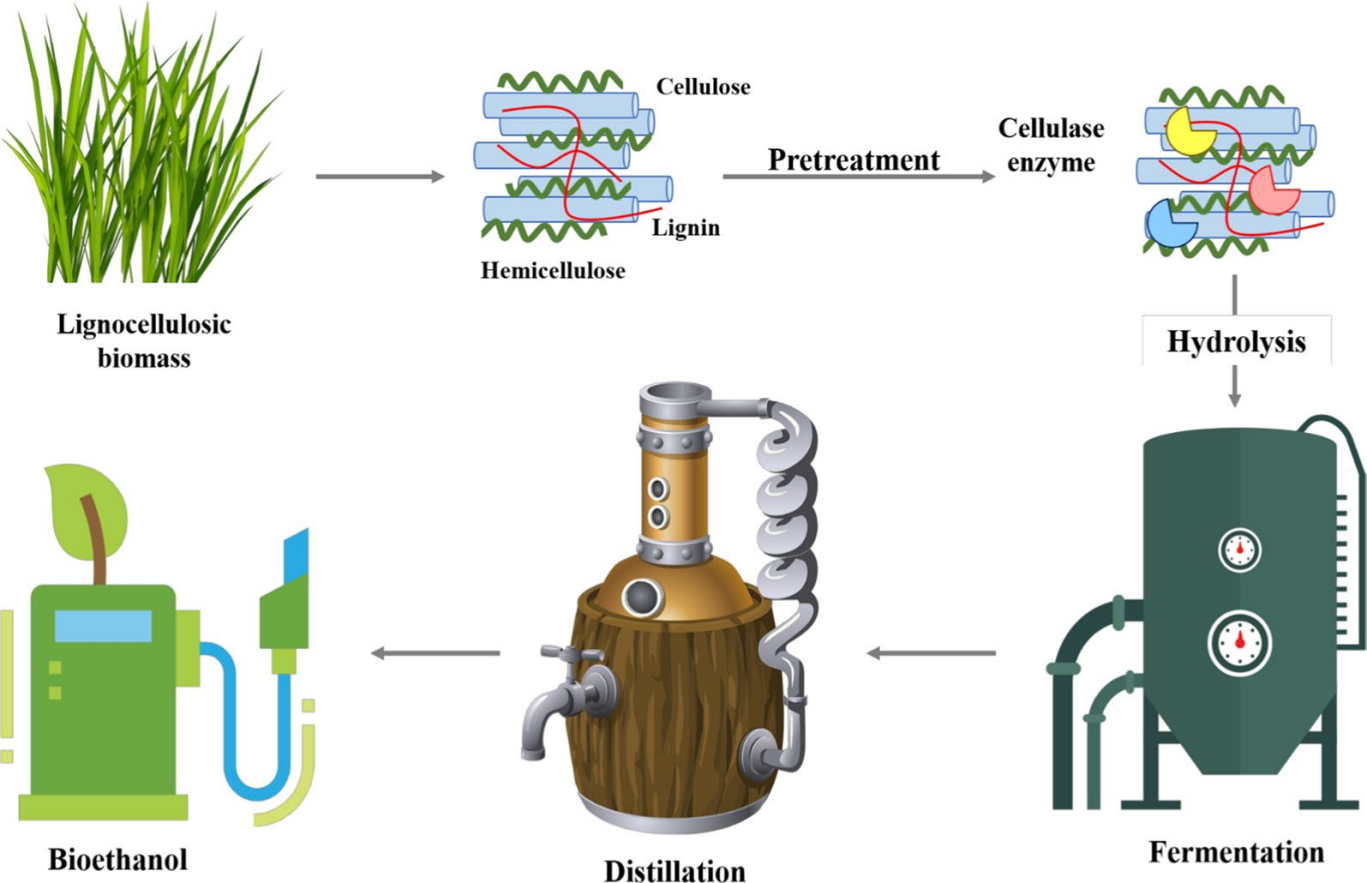
Diagrammatic representation of the production of bioethanol through various processes along with cellulase enzyme effect on lignocellulosic biomass

Pretreatment is required to disintegrate the lignin structure and to make the cellulosic complex more accessible for hydrolysis by enhancing enzyme accessibility. Pretreatment is used to reinforce the accessibility of biomass for the conversion of cellulose to glucose, thus making it more accessible to the enzymatic action by hydrolysis of hemicellulosic content and by solubilization of lignin content in the biomass [13]. Figure 3 depicts the diagrammatic representation of the production of bioethanol through various processes along with the cellulase effect on lignocellulosic biomass.

This review paper covers various pretreatment techniques with an integrated approach toward the degradation of the recalcitrant structure of biomass. Various distinct pretreatment methods are examined in this review along with integrated pretreatment approaches with emphasis on the effect of pretreatment on numerous lignocellulosic biomass. There is a huge need to produce bioethanol in a cost-efficient manner and make it available for commercial purposes. It has been earlier formulated that pretreatment and hydrolysis are relatively costlier processes. The main concern remains regarding the strategy that should be adopted to make biomass affordable for further processing towards ethanol production, which can act as a replacement for petroleum-based fuel that will further solve issues related to dependency on petroleum fuels and provide flexibility in the operation.

## Lignocellulosic biomass structure

Lignocellulosic biomass is a complex structure consisting of fermentable and non-fermentable sugar. Cellulose is the most abundant LCB (lignocellulosic biomass) with compositional analysis of 33–47% that is utilized for further process of hydrolysis [14]. Another copious compound in the LCB is hemicellulose (19–27%) in composition. Non-fermentable part of biomass are lignin (5–24%) and silica (18.3%) component which forms lignin–carbohydrates complex and hinders the further process of hydrolysis by binding with cellulose, reducing the exposed surface area for enzymatic action [15] as well as forms a hindrance against external encroachment and prevents degradation. Both hemicellulose and lignin form cover over a cellulosic portion of biomass and reduce the efficiency of enzymatic hydrolysis and fermentation which ultimately lowers the product yield. It is a prerequisite to have the region-wise analysis of biomass as LCB (lignocellulosic biomass), as a versatile resource not only used for biofuel production, but also turned out to account for the production of varied profit-based industrial products. With its high economic value, it is required to estimate the economic viability of the biofuel industry [16].

### Cellulose

The largest carbohydrate constituent of LCB (lignocellulosic biomass) is a polymer of anhydrous-D-glucose with a lengthy structural chain constituent of β-glucose monomers having an affinity with β-(1,4)-glycosidic bond and gathered together into microfibril bundle [17]. The linear cellulosic chain is associated together with inter- and intramolecular H-bonds presenting a different degree of polymerization. This H-bond forms a highly ordered crystalline region that makes it accessible for the activity of the hydrolytic enzyme [18]. Some regions in the cellulosic structure are less crystalline–amorphous regions that make it resistant to biodegradation and the enzyme can easily bind to cellulose in these regions to start the hydrolysis process. It has been visualized that feedstock with more cellulosic content is accessible for bioethanol production.

### Hemicellulose

Hemicellulose is the group of polysaccharides consisting of a short-branched chain of sugars such as arabino-glucouronoxylan, arabino-4-O-methyl-glucuronic- xylan, glucurono-xylan, arabino-xylan, and galactic-arabino-glucurono-xylan. In another word, it is the polymer edifice of both hexose sugars (D-glucose, D-mannose, and D-galactose) and pentose sugars (D-xylose and L-arabinose) and acetylated sugars [17]. It is a random structure containing five or six-carbon sugar. It is the second most abandoned polymer located in the secondary cell wall of plants.

The main hemicellulose in the plant cell wall is in the form of xylan, which gets converted into its by-product xylose in the hydrolysis process utilized for strain development in biomass [19]. Thus, acetylation frequently takes place during the biosynthesis of galactose residue and another by-product such as acetic acid formed by hydrolysis of hemicellulose which inhibits microbial growth and ethanol fermentation [15]. Thus, to inhibit the formation of by-products, required to maintain the temperature and retention time of hemicellulose degradation. Due to its branched-chain structure with a short lateral chain and low molecular weight, hemicellulose can easily be hydrolyzed [20].

### Lignin

It is a complex and large molecular structure, mainly formed by three types of monomers such as p-coumarin, sinapyl alcohols, and coniferyl, which are combined to form integrated and highly interlinked structure, has a high ambivalence, which is responsible for the hardness of structure [21]. It is barren of a sugar-based edifice having a 3D structure that possesses an alkyl-aryl bond among cellulose and hemicellulose moiety embedded in it and acts like an adhesive between them. Typically, the presence of lignin reduces the efficiency of enzymatic hydrolysis. Through electrostatic, hydrophobic, and H-bond interactions, lignin may bind enzymes, and the discharge of chemicals that are soluble lignin-derived may serve as harmful enzyme inhibitors [22].

**Fig. 4:**
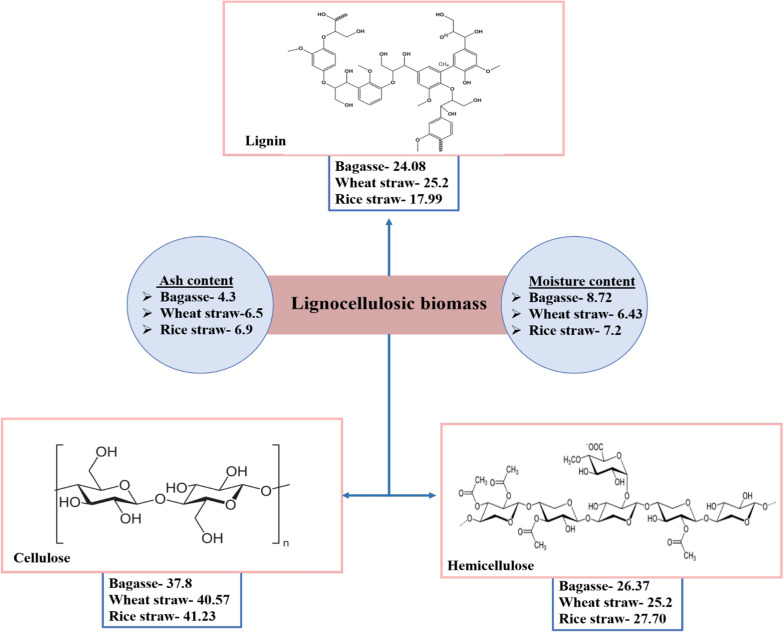
Representation of varying composition of commonly available lignocellulosic sources such as rice straw, wheat straw and sugarcane bagasse

Pretreatment processes often break down the hemicellulose polymer that links the cellulose molecules into fibres. A portion of the cellulose fibres may also be broken by pretreatment, especially in the amorphous areas. In the ensuing hydrolysis processes, the elimination of the lignin and hemicellulose improves the access of the hydrolytic reagents to the cellulose molecules. However, several physical, biological, chemical, and physiochemical pretreatment are enacted to loosen the strong interactions among these LCB (lignocellulosic biomass) and remove lignin for increasing accessibility of carbohydrates for further process of ethanol production [23]. Figure 4 depicts the widely varying composition of commonly available lignocellulosic sources such as rice straw, wheat straw, and SCB. The first step towards the utilization of LCBs is the disruption of the natural boundaries to extract the cellulose and hemicellulose, which become the substrate for the further process of saccharification. At present, this approach is to break the barrier of LCB degradation through pretreatment that can eliminate lignin and hemicellulose along with rupturing linkage with cellulose to destruct its crystalline structure and degree of polymerization [24]. It was shown that using 2% NaOH (sodium hydroxide) at 121 °C for 1 h removed the lignin content with a slight effect on cellulose and hemicellulose as compared with increasing concentration of H_2_SO_4_ in which cellulose and hemicellulose content increased while reversed with lignin content. Thus, using acid pretreatment, hemicellulose can easily be hydrolyzed [25], and further, it is required to evaluate the correct compositional analysis of lignocellulosic biomass for maximum conversion yield and to determine the economic process of bioethanol conversion. There are some methods for compositional analysis of LCBs, these are sulfuric acid hydrolysis method, kinetic analysis methods, and near-infrared spectroscopy methods [23].

The pretreatment methods show the following effect on the lignocellulosic biomass by comparing its pretreatment efficiency both before and after the pretreatment process. The pretreatment is considered to disrupt the compositional analysis of the biomass and enhances the adaptation towards available biomass with the main emphasis on particle size, and degradation of lignin, hemicellulose, and cellulose for subsequent processing. This will enhance the formation of reducing sugar and compatibility towards fermentation, further morphological analysis using XRD, TGA, FESEM, and FTIR spectra show the variation in the structural composition of biomass both before and after the pretreatment process. The efficient pretreatment has minimum sugar degradation with a slight formation of toxic compounds. The pretreatment is the pre-requisite step towards bioethanol production and its effect on feedstock are size reduction, and cellulose disruption along with hemicellulose and lignin depolarization are illustrated in the supplementary file (Additional file 1: Figure S1).

**Fig. 5:**
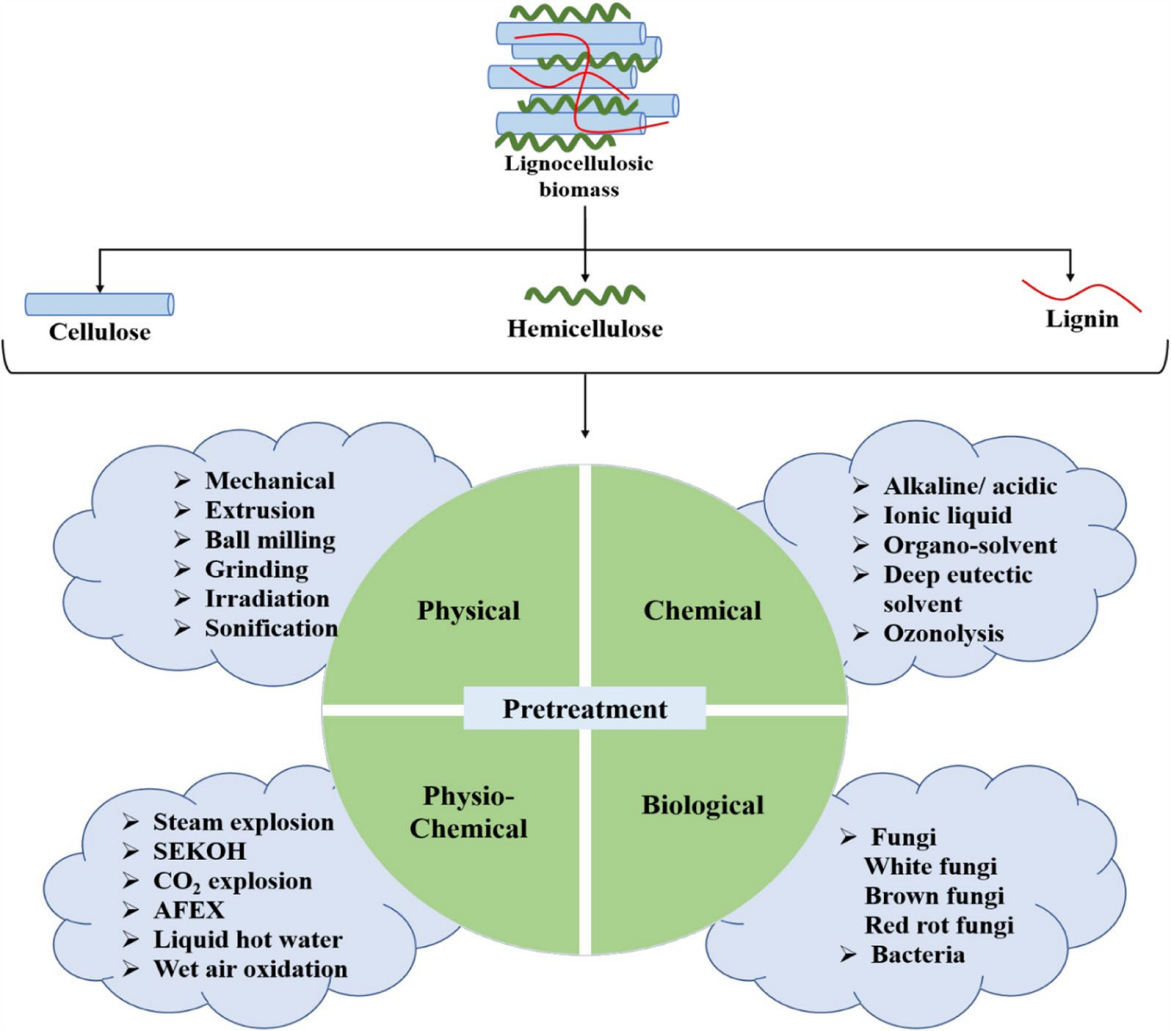
Illustration of various available pretreatment techniques performed on lignocellulosic biomass

It is quite impossible to follow the strict criteria of each pretreatment, some of the compromises can be made by associating various merits of unusual pretreatment and employing hybrid pretreatment techniques with a maximum yield of desired products. While combining these processes has increased the production cost as well as the complexity of the methods. Nevertheless, to overcome these effects, some pretreatment methods with their mechanism along with some of the hybrid forms of pretreatment methods have been discussed. The main available pretreatment techniques performed on lignocellulosic biomass, a path towards the conversion of affordable biomass available for further processing of saccharification and fermentation is depicted in Fig. 5.

## Physical pretreatment processes

Physical pretreatment mainly centres on energy and strength by disrupting the lignin barrier in the lignocellulosic complex and making sugar available for conversion to biofuels [26]. This method creates variations in temperature and pressure simultaneously requiring high energy consumption and power that results in high production costs. This pretreatment involves microwave radiation, milling, extrusion, pyrolysis, and mechanical process [27]. It is carried out through mechanized size reduction, surface area, and crystallinity index of biomass which improves further downstream processing which is an energy and cost-intensive process.

### Mechanical process

The mechanical process is the most vital step in the pretreatment process as it is meant for size reduction of the biomass along with disrupting biomass surface configuration by breaking the physical structure of feedstock. The cellulose crystallinity of agricultural waste is reduced through milling methods, which may be in the form of ball milling, wet disk milling, roll milling, grinding, and chipping processes [28]. This process enables the complete conversion of cellulose into its amorphous form and makes it available for hydrolysis so that it can easily be attacked by the hydrolytic enzyme. Ball milling pretreatment (BMP) decreases the crystallinity and size of biomass, which slackens the interior structure of biomass. It increases the internal energy of the pretreated rice straw and decreases the stability of the hydrogen bond between lignocellulosic biomasses. One of the significant importance of ball milling is that no weight loss and no inhibitor formation during the fermentation process [29]. On the other hand, high energy consumption and low effectiveness of the process have hindered its further application over other processes. Moreover, different mechanical methods have a comparable effect on biomass based on its impact, compression, friction, and shear force. It is estimated that the vibratory milling process is more effective in reducing the size of biomass as well as the crystallinity of cellulose obtained from LCBs [30]. Water absorption at 400% (w/w) during ball milling at 80 °C for 30 min on corn stover biomass enhances the glucose yield up to 66.69% than at 100 °C along with ball milling process, thus, water intake during the milling process has increased its sustainability and made the process efficient at a commercial scale [31]. Using suitable conditions for the pretreatment process by chemo-mechanical method reduces energy consumption by up to 20–80%. Thus, combining the milling process with alkaline pretreatment, i.e., NaOH proves to be the best alternative for combined process by enhancing glucose yield up to 300 mg/g of SCB biomass, the highest among various alkaline and acidic pretreatment methods. By rupturing the ether linkages in lignin/phenolics carbohydrates complexes, alkaline pretreatment can effectively remove hemicelluloses without dissolving lignin [32]. Even little dosages of dilute alkali of 4% (w/w) NaOH assisted with ball milling and then hydrothermal pretreatment at 100 ℃ for 40 min, yielded 40.75% of reducing sugar with 20.08% of xylose from pretreated SCB biomass. In this instance, NaOH had a more significant role in the deacetylation process than that of the alkaline reagent, which led to the creation of enzyme inhibitors [33]. Similarly, the combined pretreatment method of wet disk milling and liquid hot water compression at 160 ℃ for 30 min along with autoclaving at 135 ℃ for 60 min of residence time have led to 90% of glucose yield while 79% of xylose yield. The significance of using liquid hot water compression is to dissolve a portion of hemicellulose and make a portion of rice straw available for the respective action of cellulase on the surface of biomass [34]. The critical drawback of using a mechanical process for lignocellulosic conversion is that it is an energy-intensive process to break the LCB structure [35]. Due to its high energy usage in large-scale manufacturing, LCB milling increases biofuel yield but is not cost-effective. A recent study found that mechanical pretreatment improved the disintegration of structural parts in two distinct stages by decreasing the trailing duration throughout anaerobic digestion (AD) thus increasing the biofuel output by up to 22%. So, to surmount this limitation, it is required to combine both chemical and mechanical pretreatment that has been proven to lower the energy consumption of milling and ultimately increase the efficiency of glucose yield.

### Extrusion process

Nowadays, the combined process of pretreatment has increased attention. One such process is the extrusion process, which mainly includes a combination of mechanical, thermal, and chemical techniques simultaneously, leading to structural alteration due to force generated by high-power rotation. The rotation process generates shearing force among different components associated with the process, i.e., biomass, screw, and barrel, that lead to an increase in temperature and pressure of the barrel [24]. This rotation reduces biomass, efficient heat transfer, and ultimately leads to high sugar recovery from the biomass. The pretreatment process is performed radically at three reaction zone, namely conveying, reverse, and kneading. The conveying zone automatically squeezes the biomass and dispatches it to the kneading zone, where the catalyst gets mixed and forwards it to the next zone, i.e., the reverse, conveying zone where the reactor maintains the pressure required for the process. There is the continuous movement of biomass from the kneading zone to conveying zone for the pretreatment process [36]. It may be either single-gear or twin-gear extrusion-mediated pretreatment required for effective sugar recovery from various types of biomasses available. The process such as a twin-gear extruder is a promising way of pre-treating rice straw with high solid lignocellulose as it removes the amorphous region, leading to a rise in crystallinity index (CrI) up to 50% [37]. After this process, cellulose peaks become sharper, revealing an increase in glucan content ranging from 40.83% to 63.16% as well as the removal of lignin, i.e., 64.51%. Twin gear extrusion is a viable pretreatment method for lignocellulose with a high solid content in particular biomass [30]. The major optimum condition for the extrusion process is the material diameter (60 mesh), extrusion temperature (143 ℃), screw speed of 350 rpm, the reaction time of around 1.5 h; around 77.5% of cellulose and hemicellulose conversion. The findings suggest that cellulose and hemicellulose both were broken down as a result of extrusion, with hemicellulose losing more of its structural integrity than cellulose. This further suggests that cellulose is more challenging to disintegrate than hemicellulose. Additionally, it was discovered that extrusion pretreatment can result in a notable reduction in dietary fiber that is insoluble. The mechanical interruption of cell wall assembly caused by extrusion was likely caused by the combined impact of heat and shearing forces, which resulted in the breakdown of the lignocellulosic biomass structure [24]. The advantage of using the extrusion process is high continuous output, and economic feasibility, the product is obtained with no sugar degradation, under enhanced monitoring and control process with higher yield in a cost-effective manner. The aforementioned studies show that when the extrusion process combines with other pretreatment methods, it has a significant effect on the breakdown of cellulose and hemicellulose structure and enhances the total output of reducing sugars.

### Irradiation

Microwave irradiation alters the complex structure of cellulose as well as degrades hemicellulose and lignin in LCBs (lignocellulosic biomass) material and increases the enzymatic vulnerability of biomass for ethanol production. It persuades the breakdown of LCB through molecular collisions such as blending and stretching by dielectric polarization on covalent bonds between cellulose and hemicellulose. This dielectric polar movement leads to rapid heating with an elevated frequency of approximately up to a million times per second which depresses the operation time. Next, the electron beam irradiation process proved to be significant for enzyme digestibility and increases the crystalline portion of available feedstock. It is estimated that glucose yield is theoretically 52.1% higher than the untreated rice straw obtained after 132 h of hydrolysis. Due to the bombardment of electron irradiation during EBI, the interior surface of the biomass was more exposed to enzymatic hydrolysis [29]. Similarly, the researcher comprehensively studied the consequence of microwaves on chemically pretreated feedstock. This type of radiation implies 1% (w/v) NaOH pretreated rice straw yielding 31.3% ethanol in a limited time as contrasted with the traditional alkali pretreatment method [29]. The chemical treatment anticipated before microwave pretreatment disrupts the crystalline cellulose and lignin solubilization. The maximum reducing sugar yield obtained is 246.34 mg/g, cellulose content is 17.53% when pretreated with 1% (w/v) NaOH at a frequency of around 2450 MHz for 5 min at 850 W and around 150 ℃ temperature. This showed the stretching of aromatic rings (C = O bond) corresponding to acetyl groups of hemicellulose that lead to the reduction of hemicellulose content in pretreated rice straw [15]. It has been found that yield from the irradiation process increases with the decrease in the size of biomass. Similarly, the application of microwave irradiation is considered the alternative to conventional barometers as it provides a shorter heating duration with better performance, along with its immediate stop-and-start application over the feedstock. Lower dosages were probably insufficient to significantly alter glucose production. The constituents of the lignocellulose are likely to break down with the increase in irradiation doses, leading to lower glucose production [29]. The optimum condition for performing microwave irradiation is 300 W of constant power generation for 5, 10, and 15 min at 372 kPa pressure. This produces 75% cellulose hydrolysis from rye and wheat stillages. It also shows that intensive microwave pretreatment, i.e., at 372 kPa for 10 min, increases the dehydration of reducing sugar produced as well as inhibits the fermentation process, and subsequently leads to a decrease in ethanol production [38]. Thus, it is necessary to scale up the biorefinery method for the economically efficient synthesis of ethanol from microwave–alkali–acid pretreated biomass.

Another effective ϒ-irradiation of 891 kGy was appropriate for the conversion of microcrystalline cellulose with the highest degree of crack and swelling in the biomass. This is an effective pretreatment technique as the increase in the doses of irradiation has increased the glucose from 0.01% to 0.65% as well as oligosaccharide from 0.04% to 26.78%. It showed that the cellulose underwent gamma irradiation, producing carbonyl and carboxy groups at the locations of bond breaking [39]. Thus, during the microwave irradiation process, thermal "hot spots" are created that lead microwave radiation to penetrate deep into the biomass, accelerating the interaction of ions with nearby molecules of LCBs. The silicified waxy surface was disrupted and the lignin–hemicellulose matrix was broken down as a result of the dipole molecules' rapid rotation, which generated a sharp rise in temperature [33].

### Sonification

Ultrasound (sonification) is the advanced technique of pretreatment of LCBs (lignocellulosic biomass). Ultrasound-assisted pretreatment alters both the physical and chemical properties of biomass. This pretreatment process recognizes itself as an efficient and eco-friendly technique. This helps in the formation of subsequent bubbles that lead to the disruption of cellulose and hemicellulose recalcitrant structure along with an increase in the porosity of cellulosic content leading to its breakage into simple reducing sugar [15]. The deformation process of ultrasonic pretreatment reduces the lignin percentage, disrupts the biomass functional groups, and increases the crystallinity index along with an increase in the surface porosity and area of biomass. The sonification process was performed mainly at a frequency around 24 kHz and at an operating power of 400 W. Ultrasonic-assisted alkali pretreatment had been used desirably to boost the efficiency of alkali-treated biomass that can increase the lignin removal up to 80–85% and is performed with 0.5% (w/v) NaOH alkaline solution. This would consequently release more carbohydrates with fewer fermentation-inhibitory residues through the creation of Ultrasound induced cavitation [40]. Likewise, ultrasonic-assisted acid pretreatment has made its way adjacent to bioethanol production from waste potato mass. Further, it was assumed that the increase in sonification time from 5 to 10 min consequently, increases bioethanol yield up to 65.8 mg/l. Higher ultrasonic time, i.e., > 10 min disintegrates starch particles and releases the lignin in the cell wall that forbids the saccharification process and disfavors bioethanol output at a subsequent stage [41]. This ultrasonic wave of 20 kHz creates a disturbance, cavitation, or agitation in the chemistry of the lignocellulosic biomass structure. Cavitation in the biomass structure increases the mass transfer and reduction in the particle size of the exposed biomass. This process can be applied even at mild concentrations and showed regular cleavage of rice straw structure resembling rough and irregular surfaces. The microporous structure of interior biomass enables easy penetration of enzymes [42].

## Chemical pretreatment processes

This pretreatment is mainly based on the use of chemicals in the procedure which transforms the crystalline structure of lignocellulosic mass into an amorphous form in need of energy requirement. For chemical pretreatment, it is required to maintain ambient temperature during the process which subsequently enhances the glucose yield for further process. Various chemicals have different abilities to break down the compact structure of lignocellulosic biomass. These are meant to disturb and break the hydrogen bond along with the covalent bond between cellulose, hemicellulose, and lignin. Thus, it is required to understand the mechanism of the process along with the advantages and disadvantages of the particular chemical pretreatment method (Table 1).

**Table 1:** Various physical and chemical pretreatments with associated advantages and disadvantage

Pretreatment process	Mechanism of processes	Advantages	Disadvantages	Reference
Physical pretreatment				
Mechanical process	Mechanical force is required to reduce particle size as well as disrupt their surface configuration	In this process, there has been durability and robustness of the equipment used that’s why most suitable for industrial applications	Highly energy-devouring process along with slight cellulose digestibility. To enhance substrate durability there is a need for design equipment	[32–34]
Extrusion process	Extrusion process is the processing technology that includes mixing, heating, stirring, crushing and shearing of the biomass	Simple process, low energy consumption, short residence time, moderate temperature and pressure and ease of commercial usability	The amount of compressive force is very high due to which regular checking to ensure its setup, a highly time-consuming process, setup cost is very costly	[36, 37]
Irradiation	Frequency required in microwave radiation is around 0.3-300 GHz. It induces the breakdown of LCBs through molecular collision and causes dielectric polarization in a chemical bond	Shorter heating period, Moderate temperature, less formation of inhibitors	Zone with higher temperature led to the formation of furfural and HMF compounds as well as carbohydrate degradation	[29, 38, 39]
Sonification	The sonification wave is higher than 2000 Hz with the best directive and penetrating power	Eco-friendly and chemical-free pretreatment methodology	Difficulty in handling the process and parameters required for the process	[40–42]
Chemical pretreatment				
Alkaline pretreatment	In this, an alkaline solution is used to remove the uronic acid substituted on hemicellulose exterior portion that reduces the enzymes accessibility for the hydrolysis process	This process has removed the lignin fraction of mass without much sugar degradation. High efficiency and milder condition are utilized during the process	High reaction volumes and longer reaction times along with a decrease in the crystallinity, possibility of alkaline being converted to their respective salt	[44, 48]
Organosolvent	The solvents used in this process are amalgamated in water and thus operated in a homogenous system	Non-toxic, low cost, environmentally friendly as mostly are biomass derivatives and hence can easily be recycled	It can easily be evaporated due to its low boiling point	[57]
Acidic pretreatment	Using acid to solubilize hemicellulose fraction which increases the accessibility of enzyme towards cellulosic fraction and to modify lignin structure	Help in lignocellulosic matrix disruption and amorphous cellulose conversion	Acid corrosion The high reaction temperature and lower reaction time, A neutralization step is required after acidic pretreatment	[62]
Ionic liquid pretreatment	It is termed a green solvent and can disintegrate cellulose under natural conditions	High thermal stability, non-volatile and recyclable, reinforce the hydrogen bond acceptor and huge polarity, recycling and reuse facilitate it for reducing cost	High cost, sufficient towards removing lignin and hemicellulose avoids its use in a broad variety of applications	[68, 71–73]

### Alkaline pretreatment

Among chemical pretreatment methods, alkaline pretreatment was widely accepted as being a simple process and having a strong pretreatment effect over some time. This method selectively removes lignin from its carbohydrate counterparts and expands the surface area as well as the porosity of the biomass, decrease in the polymerization degree and crystallinity, resulting in enhancing enzymatic hydrolysis [21]. The chemicals used under alkaline pretreatment are non-corrosive and non-pollutants such as sodium hydroxide (NaOH), ammonia and lime (calcium hydroxide, Ca(OH)_2_), sulfite, and ammonium hydroxide [43]. The common effect regarding alkaline pretreatment is that it raises the digestibility of lignocellulosic biomass, which can be obtained by transforming the intricate lignin–hemicellulose network enhancing lignin removal. With rupturing cellulose's H-bonds and enabling amorphous cellulose more soluble at higher temperatures or longer residence times, high-severity conditions cause the thermochemical alterations [44]. Experimental analysis reveals that NaOH is the most effective pretreatment which breaks the intercellular bond between cellulose and another component (lignin and hemicellulose). NaOH pre-treated biomass causes the lignin to break down by solubilizing the lignin carbohydrate bond and increases the surface area of cellulose structure while minimizing the degree of crystallinity and polymerization, carried out at low temperature and pressure [45]. In alkaline conditions, lignin's alkyl-aryl bonds are easily disrupted for enzymatic activity.

In a comparison of various alkaline pretreatment processes, alkaline peroxide is best as it increases the fermentation yield by solubilizing lignin effectively and increases the digestibility of feedstock required for further process. Alkaline peroxide used for the process was 5% H_2_O_2_ solid concentration at 50 ℃ for 1 h performed on rape seed straw which results in higher enzyme digestibility [46]. This alkaline peroxide treatment before alkaline pretreatment is performed at mild conditions effectively destructing the structure of biomass while facilitating isolation of lignin from the complex recalcitrant structure of the macromolecules. The peroxide loading carried out at 80 mg/g of pretreated wheat straw results in 59.9% lignin removal due to the degradation of lignin during the process [47].

Alkaline peroxide pretreatment is combined with other two oxidizing reagents namely NaOH and HCL (37%) with H_2_O_2_ (33%) employing different thermo and thermochemical reactions by autoclaving both reagents used in the process. This shows around 74% hemicellulose solubilization with the release of 2.6% of glucose as compared with pretreatment methods performed alone. Since NaOH shows strong lignin removal and hemicellulose solubilization by breaking ester linkages, it is frequently used for lignocellulose pretreatment. This increases biomass porosity and deteriorates the polysaccharide chain and cellulosic content [48]. Similarly, pretreatment with 1% NaOH assisted with 1% of H_2_O_2_ treatment for 24 h results in total sugar of 0.17 g/g of dry biomass while reducing sugar is about 0.024 g/g of dry biomass after 48 h at ambient temperature. Furfural and other inhibitors of hemicellulose breakdown were not taken into consideration with most alkaline solutions perturbing and disintegrating the association of LCBs structure but do not destroy hemicellulose like acid pretreatment. Following alkaline pretreatment, the diversity of sugars found in the liquid fraction shows that NaOH can absorb a wide range of soluble compounds under moderate circumstances [49]. Likewise, NaOH catalyzed organosolvent pretreatment with 10% loading of NaOH along with 150 ml of ethanol/water at 60/40 (%v/v) ratio at 180 ℃ for 30 min resulted in the solid recovery of 81.2% along with lignin removal of 40.7% from SCB biomass. However, when the alkali concentration rises, more cellulose can be converted into oligosaccharides and subunits, reducing the amount of solid recovered and the amount of sugar produced [50]. Further another process of NaOH along with a hydroxymethylation process using 20% (w/w) in the ratio 1:1.5 (w/w), resulted in lignin reduction of 12.46% and enhances the glucose yield up to 70.33% after 3 h of incubation time. For the partial replacement of phenol in the manufacture of phenol–formaldehyde (PF) resins, hydroxymethylation was employed in SCB biomass. It can increase susceptibility and lignin’s hydrophilicity by introducing hydroxylic units into the ortho regions of aromatic rings. The key mechanism thought to be responsible for the ineffective adsorption of the enzyme to the lignin in the hydrophobic part [51]. Another alkaline pretreatment using 20% (w/w) KOH at 120 ℃ for 40 min shows 88.2% of delignification from wheat straw biomass. In addition to making cellulose more accessible, lignin removal also lessened the significant lignin–enzyme adsorption that occurs during enzymatic hydrolysis. With more KOH being used, lignin removal improved as well as glucan degraded more slowly than xylan, which was a result of cellulose's substantial polymerization rate and crystallinity. As a result, under the alkaline pretreatment, cellulose was more persistent than hemicellulose [52]. In the case of KOH/urea (KU) pretreatment at low temperature (< 100 ℃) pre-treated rice straw with 3% along with a solid-to-liquid ratio of 1:15 for 3 h at 70 ℃ temperature. The estimated sugar yield was about 92.38% with an obtained glucose concentration of 24.04 g/l. The combination of alkali/urea has a stronger effect on the composition of rice straw's cell wall as compared to solitary KOH pretreatment. As a result, permeability, fiber stratification, and delignification were all considerably enhanced by the combination of KU, which was 18.11% greater than the effects of KOH pretreated alone [53]. Similarly, on corn stover, mild KU treatments in the ratio 1:1 with 2% w/v have increased glucose yield up to 83.1% and sugar yield up to 6.3 folds. By successfully cleaving the chemical bond among both polysaccharides and lignin, urea might remove some lignin and make it easier for KOH to remove the remaining lignin [54].

In addition to the above alkaline pretreatment, an effective pretreatment technique using 0.5 M Na_2_CO_3_ at 93 ℃ for 3 h has resulted in 153 g of ethanol from 1 kg of biomass. In the Na_2_CO_3_ solution, silica (SiO_2_) is changed into sodium silicate. In the alkaline media, sodium silicate dissolves as a result of an endothermic process, resulting in 95% of the ash removal from the rice straw. The amount of lignin, porosity and exposed surface area of the biomass all have an impact on the rate at which cellulase binds to it [7]. An alternative process of mixed alkali soaking methods in 0.5% K_2_CO_3_ or 0.068% K_2_SO_3_ that is autoclaved at 120 ℃ for 40 min resulted in 32.94 g/l of reducing sugar along with 20.18 g/l of glucose content. Thus, sulfite (SO_3_^2−^) is reported to be able to cleave α-alkyl ether links, α-benzyl ether linkages, and β-benzyl ether couplings on phenolic lignin units along with sulfonation of lignin, improving the enzymatic saccharification. Lignin and different uronic acid replacements in hemicellulose can be eliminated by carbonate (CO_3_^2−^). This technique was considered a one-pot pretreatment process with less loading of alkali salts [55]. High pressure at 130 kPa assisted with alkali (2.5% NaOH) pretreatment for 40 min enhanced the cellulose content in the biomass up to 64.07% which further increased with the rise in pressure. The maximum conversion of cellulose at 45.82% is due to the rupturing of ester bond crosslink between lignin and xylan that results in maximum delignification as well as biomass porosity [56]. Thus, alkaline pretreatment is believed as most efficacious among all chemical pretreatment procedures. The main limitation of this pretreatment includes a relatively long pretreatment time as well as high alkali consumption.

### Acid pretreatment

Generally, acid pretreatment is brought out either by diluted or concentrated acid, still, dilute acid is preferred over concentrated one due to its non-corrosive property. The concentrated acid on the other had shown a specific mechanistic hindrance, during the pretreatment process where the cellulose obtained from the feedstock releases a catalyst similar to a proton that cleaves the heterocyclic ether linkages between polymers and chain monomers of hemicellulose. With this cleavage, some monosaccharides settle along with it, allowing cellulose to have less accessibility to the enzymatic action during the hydrolysis process and showing little degradation towards lignin. Thus, using dilute acid for chemical pretreatment is effective, in enhancing the hydrolysis of amorphous cellulose and solubilization of hemicellulose [57]. The diagrammatic representation showing the effect of acid pretreatment on feedstock is illustrated in the supplementary file (Additional file 1: Figure S2). The essential acids used for pretreatment are sulphuric acid, hydrochloric acid, phosphoric acid, nitric acid, etc. Among various acids used for pretreatment, sulfuric acid is considered better because it is tremendously efficacious and economical in nature [58]. Likewise, a pretreatment technique with 79.6% (w/w) phosphoric acid assisted with 1.9% (w/w) hydrogen peroxide (PHP) with a solid–liquid ratio of 1:10 (w/w) that is carried out in a rotatory shaker at 180 rpm for 2.9 h at 40.2 ℃ has resulted into 16 g of ethanol production. These results showed that the PHP pretreatment was extremely effective at depolymerizing lignin; moreover, substantially less molecular weight and chemical compositions of PHP lignin being more homogeneous will become more suitable for any potential applications [59]. Additionally, this process recovers concentrated phosphoric acid, treated phosphorus, and lignin from the pre-treated sample using 74.92 g (85% w/w) H_3_PO_4_ and 5.08 g (30% w/w) H_2_O_2_ with 96.3% conversion of cellulose to glucose. To speed up the oxidation-based cellulose degradation in PHP pretreatment, the produced organic acids from lignocellulosic fractions, such as acetic acid and formic acid, would collect and may readily form additional peroxyl acids. However, the deteriorated lignocellulose fractions, particularly the lignin fraction, will contribute to the formation of phosphate ionic liquids, which would increase the depolymerization of cellulose and lead to a reduction in cellulose recovery [60]. The effective concentration of acid for pretreatment is generally down to 4%, in these reactions, and for determining the yield of reducing sugar in this procedure liquid fraction is used, while in cases of enzymatic hydrolysis, a solid fraction is dried and utilized [57].

Another process of chemical impregnation is used for reducing the energy consumption during the refining process which is carried out using 0.2 M NH_4_Cl impregnation for 10 min along with a temperature of 170 ℃ which results in 97.4% of xylose recovery with less degradation of cellulose along with solubilization of lignin and hemicellulose leading to an increase in the cellulosic content to 66.11%. This high temperature caused NH_4_Cl to break down quickly into NH_3_ and HCl which increases the acidity of aq. solution and speed up the breakdown of cellulose and hemicellulose [61]. The main intention of cellulosic ethanol production is efficient or cost-effective biomass pretreatment with a maximum yield of bioethanol. Thus, microwave-assisted acidic pretreatment along with cellulolytic enzyme conversion is the alternative pretreatment for conventional production as it ensures a high degree of cellulosic degradation [62]. High temperatures generally make cellulose fibrils more prone to breaking, this also speeds up the process by which simple sugars are transformed into inhibitory substances. The parameters of effective microwave pretreatment are its exposure for 15 min and applied pressure of around 372 kPa which gives a cellulosic yield of up to 75% as compared to 1048 kPa pressure with similar time exposure, leading to a slump in yield of cellulosic hydrolysis of up to 28%. Eventually increasing pressure and exposure time affects the degradation of sugar along with a decrease in galactose, arabinose, and xylose concentration and an increase in the formation of inhibitors such as furfurals [63].

Subsequently, chemical pretreatment assisted with microwave is considered as efficient for the recalcitrant structure of biomass. The chemical used for the process includes CoCl_2_, NiCl_2_, and CrO_3_ at 0.25 M concentration that is kept in a microwave vessel, after soaking for 12 h with 3% orthophosphoric acid blended with the mixture. In this CoCl_2_ act as the Lewis acid when dissolved in water, giving high acidic strength that improves the pretreatment efficiency [64]. The resultant mixture is kept in a microwave reactor of 700 W magnetron and the maximum delignification of 52.8% is shown by CoCl_2_ assisted with H_3_PO_4_ while maximum ethanol yield is obtained by microwave-assisted NiCl_3_ + H_3_PO_4_ [65]. The aqueous solution in which the metal salts are present acts as a Lewis acid, giving it a strong potential for electron attraction that allows it to dissolve glycosidic links in hemicellulosic sugar chains. Due to its quick response rate and little preparation time, microwave heating can start and stop reactions instantly. While 0.3 wt.% H_2_SO_4_ pretreatment catalyzed with 80% acetic acid at 107 ℃ for 1.5 h has reduced the lignin content up to 12.3% from the pretreated sample as compared to 24.7% from untreated biomass with a delignification rate of 71.6% from the biomass. This is due to the acylation of hydroxyl group of the biomass [66]. This would ultimately reduce the activation energy with the addition of acid along with metal salts. Further, the pyrolysis of metal salts leads to the creation of metal base nanoparticles that serve as the in situ catalysts for the formation of bio-based products. However, some limitations as acid revival are a little bit costlier as compared with other pretreatment methods, the formation of inhibitors and a comparatively higher temperature are required for the conversion of glucose to cellulose.

### Ionic liquid pretreatment

In recent works, ionic liquid pretreatment has attracted concern due to its ability to disintegrate cellulose at ordinary conditions and has resulted in maximum output of sugars and carbohydrates. Moreover, it tends to remain in its liquid form in a broad series of temperatures. It is also termed a green solvent with high thermal and chemical stability. The ionic liquid is considered a non-volatile liquid, non-flammable, and recyclable in nature which helps in reducing processing costs [67].

Several types of ionic liquids (ILs) such as imidazolium-based ([(C_3_N_2_) Xn]^+^), pyridinium-based ([(C_5_N) Xn]^+^), pyrrolidinium-based ([(C_4_N) Xn]^+^), ammonium-based ([NX_4_]^+^, phosphonium-based (PX_4_]^+^, and sulfonium-based ([SX_3_]^+^ ILs, have been used for pretreatment of LCBs. Acidic ILs have more potential for LCB pretreatment as they have an impressive outcome on the depolymerization of lignin by rupturing ether bonds to disrupt the lignin structure [21]. It has dissolved various polysaccharides including cellulosic content in the biomass using pyridinium chloride as an acidic ILs that has disrupted the recalcitrant structure of biomass by forming strong hydrogen bonds and creating space for penetration of solvent [68]. The creation of electron donor–electron acceptor complexes between the inherent hydroxyl groups of cellulose and Ionic liquid would cause pure cellulose to dissolve in ILs. Its inter- and intra- H-bond network may be disrupted as a result of these interactions during pretreatment, which would increase enzymatic digestibility [69]. It is estimated that a concentration of ionic liquid of more than 10% (v/v) deactivates cellulase even for the highly complex structure of biomass. The aqueous Ionic liquid, i.e., 1-ethyl-3-methylimidazolium methyl phosphonate ([C2mim][MeO(H)PO_2_]) is considered as more congruent for enzymatic action, used at a concentration up to 30% (v/v) which shows synergistic alacrity of cellulase diversification using aqueous ionic liquids [70]. Some other advantages include its environmentally benign, recycling efficiency at over 99% which makes the process effective and have superior cellulose solubilizing characteristics. However, these ILs including Cl^−^ and OH^−^ are corrosive in nature which adversely affects enzyme activities resulting in lower hydrolysis yield. Using 1-ethyl-3-methylimidazolium acetate [EMIM][OAc] at 120 ℃ for 5 h is considered an alternative to enhance the saccharification process with the improved ethanol yield of 75% that is due to more accessible morphology, less crystallinity with lower lignin and hemicellulose concentration [71, 72]. The production of bioethanol is greatly dependent on the pretreatment process, by combining two methods with optimized enzymatic conversion of pretreated biomass. Polyethylene glycol (PEG) assisted with IL [1-butyl-3-methylimidazolium chloride (BMIMCl)] at 5% (w/w) results in maximum enzymatic conversion and bioethanol yield [71, 73]. This combined study demonstrates that with an increase in PEG concentration and reaction time has decreased the composition of both glucan and xylan. The reducing sugar yield of 62% at an optimum condition of 154 ℃ temperature and 5% (w/w) PEG for 60 min yielded bioethanol production of around 84% in juxtaposition with the biomass pretreated alone by IL. Because cellulose contains more hydrogen bonds than the remaining components in biomass, adding an anti-solvent causes more water molecules to replace IL in H-bonds, which increases the precipitation of the cellulose part of the lignocelluloses [74]. Similarly, using surfactant at different concentrations shows a higher concentration of surfactant has enhanced bioethanol yield subsequently. The highest glucose yield of 176 g/l is obtained after 72 h of pretreatment using PEG at 5% concentration with an ethanol yield of about 105.4 g/l. Thus, it is estimated that the concentration of PEG has a significant role in efficient ethanol yield as it acts as an antibacterial agent that promotes optimal yeast activity [75].

### Organosolvent

An aqueous organic solvent like glycerol, acetone, methanol, etc., is added to the biomass underlying, with distinct temperature and pressure conditions that occurred in the presence of acids, bases, or catalysts [76]. The catalysts utilized during the process involve H_2_SO_4_, NaOH, and MgSO_4_ assisted with organosolvent. H_2_SO_4_ is considered the most effective catalyst for maximum ethanol yield as it is a sturdy chemical, but is highly toxic, corrosive, and has inhibitory properties. NaOH is another effective catalyst with a concentration of 2% (w/w) or more. Among various organosolvent, glycerol is preferred over others as it is a non-lethal, easily accessible solvent at a more affordable rate with a high boiling point of 290 ℃. Recently, it is reported that adding water at a lower concentration, i.e., 10% with glycerol can reduce biomass viscosity at higher solid loading, leading to excellent glucan digestibility. Another effective pretreatment technique using glycerol at a higher concentration of 60% at a temperature of 190 ℃ for 60 min resulted in higher cellulose accessibility of up to 89% due to the removal of hemicellulose and lignin, this ultimately increases saccharification yield [77]. Because xylan and lignin were removed, the cellulose digestibility was greater in the biomass that had been processed with glycerol. These findings demonstrated that lignin and xylan both served as cellulase barriers [78]. Due to its non-productive and irrevocable adhesion of cellulases and the development of a protective coating over cellulose fibres that restrict cellulose exposure, lignin is the main inhibitor of cellulases. It is also known that the impact of xylan is caused by cellulase being inhibited by xylooligomers produced during enzymatic hydrolysis [77]. Similar to glycerol, glycerol carbonate is also used with a comparatively more boiling point than glycerol, i.e., 354 ℃. Following pretreatment using glycerol, a solvent that may reduce the biomass surface tension at elevated temperatures and may aid in the generation of the enzyme–substrate complex. This may account for the rise in substrate-specific hydrolytic activity that led to an improvement in glucan digestibility [79]. The novel combined pretreatment method of 50% (v/v) ethylene glycol assisted with 2% H_2_SO_4_ used as a catalyst has resulted in maximum delignification of 48.2% while 95.8% of cellulose recovery is obtained within 15 min at 150 ℃ temperature. It is also concluded that the increase in ethylene glycol concentration encourages high lignin liquefaction [80]_._

### Deep eutectic solvent

Another pretreatment approach related to organosolvent is the deep eutectic solvent that includes both hydrogen bond donors and acceptors with relatively low toxicity as well as biocompatibility. The increased size of choline chloride (ChCl) pretreated biomass may be the consequence of more hydroxyethyl or methyl groups, which decreases the lignin removal by increasing viscosity and steric hindrance [81]. Combined method of ChCl/glycerol and sorbitol in the ratio 1:10 assisted with an ultrasonic wave of frequency 20 kHz with the emission power of 40 W for 20 min retention time results in a reducing sugar yield of about 276.8 mg/g from SCB. The rise in the consistency of the reaction, which may have persisted after the appropriate biomass pretreatment due to insufficient mixing and mass transfer, is the likely cause of the loss in sugar yield that occurred when the mole fraction of ChCl/sorbitol was increased [82]. A similar deep eutectic solvent assisted with diluting NaOH at 0.75 wt.% and pre-soaking in lactic acid for 40 min at 140 ℃ temperature resulting in 62% of xylan removal and 49% of delignification. The biomass recovery considerably dropped due to enhanced delignification, and the output of reducing sugars steadily increased. According to the aforementioned data, lignin removal increased the efficiency of enzymatic saccharification [83]. Apart from choline chloride or monoethanolamine, the maximum cellulose digestibility approximately 228% higher is exhibited by triethyl benzyl ammonium chloride assisted with lactic acid with solid-to-liquid ratio maintained at 1:15 for 4 h at 120 ℃ temperature with lignin degradation ascribed at 88.72% and xylan removal at 73.93% along with 88.23% of cellulose digestibility. Thus, high temperature may increase the bond rupture between cellulose, lignin, and xylan, making it easier to selectively extract lignin and increasing the effectiveness of enzymatic hydrolysis [22]. Thus, DESs are regarded as more ecologically acceptable and sustainable solutions than ionic liquids due to their good physicochemical properties, cheap cost, low toxicity, and biodegradable properties (ILs). Furthermore, DESs preparation is simpler and less expensive than ILs, and it necessitates less sophisticated purification processes [84].

## Physiochemical pretreatment processes

This pretreatment is a mixing of both physical and chemical processes in a single pot. Different pretreatment strategies used under this process are steam explosion, wet air oxidation, ammonium fibre explosion, CO_2_ explosion, and liquid hot water process that have a wide range of applications on various feedstock with their ability to enhance the production of sugar along with the maximum level of delignification. These processes are considered relatively environment-friendly processes meant for extracting hemicellulose from lignocellulosic biomass and altering the structure of cellulose for further processing. Various processes involved during physiochemical pretreatment with their relevant temperature and pressure required to carry out the process along with the composition of reducing sugar obtain after the process are illustrated in Table 2.

**Table 2:** Comparison between various physiochemical methods

Types	Temperature (T) and pressure (P)	%Yield of sugar	Advantage	Disadvantage	References
Steam explosion	T: 180-280 ℃ P: 2.5–7 MPa	Glucose recovery 57–63%	Simple biomass pretreatment	- Generate toxically compounds - Disturb the sustainability of enzymes	[20, 85]
Liquid hot water	T: 160-240 ℃ P: > 5 MPa	Glucose recovery 73.1%	Simple process Low capital Low maintenance costs Non-corrosion problem High sugar yields	Large amounts of energy are required due to high water consumption	[93, 100]
CO_2_ explosion	T: 190 ℃ 20–60 bar CO_2_ pressure	Glucose yields 80.7%	Cheaper Higher yield Low-temperature requirement	Not relevant for biomass with less moisture amount	[104, 109]
AFEX process	T: 60-100 ℃ P: 1.7–201 MPa 27% (w/w) ammonia concentration	42% lignin reduction	Reduce crystallinity and amorphous structure of cellulose	High energy is required to maintain process temperature	[105]
Wet air oxidation	T: 170-200 ℃ P: 10–20 MPa	67% Cellulose content, 89% lignin removal,	Simple method Suitable for lignin-enriched biomass residues	High cost to maintain it, cellulose is less affected	[113]

### Steam explosion

Steam explosion pretreatment is similar to the autohydrolysis technique, which is one of the basic and widely accepted physio-chemical pretreatment methods, due to its environment-friendly nature. It is widely considered a highly cost-effective option over other pretreatment methods. In the steam explosion process, the lignocellulosic biomass (LCB) is promptly heated with saturated steam at high pressure for a short period, probably for a minute, followed by the sudden release of pressure causing expansion of steam within lignocellulosic material that results in the detachment of individual fibres by interrupting the cell wall structure and solubilizing hemicellulose and lignin [20]. It is the most widely used method, as it affects the physiochemical properties of lignocellulosic biomass (LCB) by breaking down lignocellulosic structure and hydrolyzing the hemicellulosic fraction with the lesser environmental consequence, along with high energy efficiency. Major drawbacks associated with it are partial lignin removal and the production of some toxic chemicals such as acetic acid, furfural, and hydroxymethylfurfural. During the process, inhibitory removal is required as it disturbs the sustainability of enzymes during hydrolysis and fermentation which sometimes results in high production costs [21]. This pretreatment method is performed at 689 kPa pressure and a high temperature of 160–260 ℃ in different residence times, i.e., 15, 30, and 60 min, along with sudden depressurization. It was evaluated that the total sugars were less concentrated when the material was exposed to the pretreatment for longer periods. As a result, applying SE increased the quantity of total sugar accessible in comparison to such material that had no prior treatment. Therefore, it indicated that the operation of steam to dissolve the cellulose's protective hemicellulose and lignin structure was adequate [57]. It is evaluated the protective effect of hemicellulose and lignin on cellulose as well as the strength of the ether bonds (α-O-4, β-O-4, 4-O-5) and C–C bonds of the lignin might be used to explain alteration in the cell wall composition of the LCB. To increase the performance of the steam explosion process, acid or alkali impregnation is generally utilized before pretreatment, which increases the cellulose digestibility and solubilizes a particular portion of hemicellulose which ultimately increases recovery of sugar.

Acid catalysts such as SO_2_ and H_2_SO_4_ are used as impregnating agents which decreases the temperature and time required for sugar recovery as well as enzymatic hydrolysis of steam-pretreated biomass. Maximum yield is obtained at a specific temperature of 180 ℃ and time for 10 min using 0.9% (w/w) of dilute H_2_SO_4_. But in harsher circumstances, more solubilization was attained, demonstrating the significant impact of acid addition as well as residence time on solids extraction. It shows that under extreme circumstances, SE causes the cellulose fiber to first breakdown, which results in a reduction of glucose in the solid residue [85]. Acid-catalysed steam explosion using 0.25% (w/w) H_2_SO_4_ with pressure at 103 kPa and 121 ℃ for 60 min resulted in 34.08% of cellulosic content. Thus, an increase in the concentration of acid results in a decrease in the cellulosic content of the biomass due to the formation of inhibitors [86]. Similarly, acid-impregnated steam explosion process maximizes the efficiency of saccharification for hemicellulose and cellulose which enhances the formation of xylose-rich hydrolysate and was performed either soaking in water or by treatment with 0.5% (w/w) H_2_SO_4_ or 0.5% (w/w) H_2_PO_4_ for 10 min at 200 ℃. This leads to increases in sugar concentration to 20.4 g/l with the increase in the severity factor toward the steam explosion. This is because, under acid-catalysed circumstances, the transition of xylose to furfural begins to proceed with greater severity [87]. When the severity of steam explosion is kept between 2 to 3 the yield of xylose is below 70% while increasing severity beyond 3 has led to degradation of xylose to furfural and the cellulose formation also decreases accordingly. The optimal severity factor for the steam explosion is noted at 3.643 which maximizes the yield of xylose to 87% along with 85% of cellulose conversion. This conversion occurs due to the elimination of hemicellulose forming the complex structure of lignocellulosic biomass (LCBs) and also as xylooligosaccharide residues do not hinder the formation of cellulase enzyme. This has been evaluated that stem explosion leads to enhancing the porosity in the biomass structure and makes cellulase accessible for further processing of enzymatic hydrolysis [88]. The diagrammatical representation of combined pretreatment using chemicals along with the steam explosion process is depicted in the supplementary file (Additional file 1: Figure S3).

Additionally, the combined process of a steam explosion at 212.3 ℃ for 5 min along with the incorporation of green liquor solution prepared by stirring Na_2_S and Na_2_CO_3_ at 31% (w/w) for 28 min results in 49% of solid recovery from the bamboo biomass, used for bioethanol production that results into 62% of cellulose accessibility, demonstrating that the combination SE and ionic liquid pretreatments mostly eliminated the chemical linkages connected to the aromatic ring frameworks of lignin. On the other hand, the combined pretreatments carried out in this study ought to be the cellulose-benign technique maintaining cellulose in the pre-treated biomass residues, as the absorption spectra of β- glycosidic linkages and inter-H-bonds in polysaccharides were comparatively increased [89]. To enhance the outcome of the process, a technique using steam explosion with a semi-continuous pre-pilot reactor builds up the pressure periodically at an interval of 5 s with a maximum temperature of 200 ℃ for approximately 10 min, this subsequently increases reaction severity and thereby increases the cellulose content of the specific biomass [90]. Moreover, alkaline sulphonation before the steam explosion at 210 ℃ for 5 min enhanced the carbohydrate recovery, along with efficient ingestion of enzyme due to the maximum removal of lignin at 69.37% from the pre-treated biomass and improved hydrolysis yield to 57.89% of the biomass. This might be due to substrate acid groups lost during steam pretreatment, and this loss increased with pretreatment intensity. The loss of functional groups in the structure of lignin might be caused by increasing the severity of autohydrolysis, such as by steam pretreatment and liquid hot water pretreatment, which would accelerate the acid-catalysed depolymerization processes of lignin [91]. Similarly, the process of combined pretreatment using KOH-assisted steam explosion, i.e., SEKOH at 2% (w/v) autoclaved at 121 ℃ for 45 min has enhanced cellulose accessibility of biomass with maximum delignification of 85% along with less sugar loss during the process. The temperature utilized in this study was tested at particular intervals to extract the greatest amount of carbohydrates utilizing chemical pretreatment techniques. Additionally, KOH produced the most effective chemical pretreatment and it is utilized after a steam explosion to increase delignification [92].

### Liquid hot water pretreatment

This process is almost similar to that of steam explosion as it requires highly pressurized hot compressed water to perform the pretreatment protocol. Water is considered the exclusive solvent under a pressurized state along with high temperature [63]. Hot water is used to maintain lignocellulosic biomass (LCBs) liquid form, to prevent its degradation in the temperature ranges from 160 ℃ to 250 ℃ with a heating rate of 20 ℃/min at residence time ranging from 5 to 20 min [21]. A hydrotropic solution is a greener solvent as it is water-based and hence is safe to handle along with the higher level of hemicellulose solubilization in acidic conditions due to hydronium ion formation, which occurs due to ionization of water, leading to the release of acetic acid from hemicellulose which results in higher pentose yield in the liquid phase, more lignin removal in the solid phase along with glucose revival from enzymatic hydrolysis. Liquid hot water pretreatment assisted with sodium carbonate at 16% (v/v) along with oxygen at 0.8 MPa led to maximum hemicellulose removal and lignin removal of up to 71.4% with bioethanol concentration achievement at 66.5 g/l. Na_2_CO_3_ dissolution in water during LHW treatment produces a slightly alkaline solution, that causes partial lignin dissociation by breakage of b-aryl ether linkages and phenolic a-aryl bonds [93]. Another combined pretreatment process is liquid hot water which was performed at 180 ℃ with mechanical refining in the form of a disk milling process with low serenity of 3.36 and a maximum glucose recovery of 95.8% from SCB. After the pretreatment process, the ash content decreases from 3.96% to 2.47%, and the cellulose content increases from 37.4% to 41.7%. This was predicted since more intense LHW treatments would produce more oligomer and monosaccharides, inorganic compounds (such as acetic acid), as well as metabolic byproducts [94]. By increasing the extraction time of LHW pretreatment from 45 to 90 min, the destruction in the biomass structure occurs with maximum xylan recovery at 54%. It is known that during LHW extraction, acetyl groups connected to hemicellulose and lignin would undergo hydration to create acetic acid, which would cause the liquid to become acidic and cause hemicellulose to dissolve on its own [95]. Similarly, another combined pretreatment of liquid hot water assisted with NaOH and O_2_ at 0.6 MPa for 60 min was performed and the concentration of glucose yield was observed to be 52.2% after 40 min of residence time. Hot water-degraded acetyl groups in xylan were utilized to produce acetic acid. For the creation of acidic compounds, the hemiacetal link was broken. When water ionized at high temperatures, the pH of the water changed to an acidic state, which led to enhanced dissociation of xylan and enlarged pores in cell wall reed biomass [96]. Similarly, the increase of hydrothermal temperature from 120 ℃ to 200 ℃ has increased the cellulosic content from 41.23% to 52.62% while hemicellulose gets broken down into water-soluble saccharides and various other molecules [97].

Auto-catalysed process assisted with NaHCO_3_ at 18% (w/w) concentration at 689 kPa oxygen pressure for 12 h, along with the presence of LHW (liquid hot water) at a temperature around 210 ℃ for 10 min, resulted in 33% of lignin removal with hemicellulose recovery up to 63.47% demonstrating enhancement in the accessibility of cellulose [98]. Apart from it, hot water pretreatment at 200 ℃ for 120 min engaged with 15 ml of 2-methyl tetrahydrofuran and 1 mol/l of oxalic acid along with microwave irradiation heating at 180 ℃ for 20 min leads to an increase in hydrolysate yield up to 92.89% with a rate of cellulose conversion augmenting by 6.7 folds times of untreated biomass [99]. The advantage of dealing with liquid hot water (LHW) is the omission of expensive and hazardous chemicals as compared to other pretreatment methods, this reduces corrosion of equipment and avoids the need for solvent recycling as hot water is the main component for this pretreatment, making it a cost-effective, simple and eco-friendly process. Apart from that, disadvantages include the requirement of high energy along with excessive water as water is the sole solvent used in this method, the insignificant concentration of sugar generation as well as the formation of inhibitors such as furfural compounds [100].

### Ammonium fibre explosion process

Ammonification includes processes viz., soaking in aqueous ammonia (SAA), ammonium fibre explosion (AFEX), extractive ammonia (EA), recycled aqueous ammonia expansion, low-moisture anhydrous ammonia [101]. All of these are advanced ammonia loading-based pretreatment that has a certain impact on the composition of the cell wall with diminishing effect on cellulose crystallinity and alteration in the crystal edifice, changes in the hemicellulose and lignin structure along with morphological, physiochemical, and ultrastructural changes in the biomass cell wall [102]. This aqueous ammonia is an inexpensive solvent that interacts with the C–O–C bond and breaks both ester and ether bonds between cellulose and hemicellulose in the lignocellulosic structure [103]. SAA is the earliest form of the ammonia loading process in which maximum processing time and high consumption of both chemicals and water are required during the process. To overcome such a problem, the combined method of H_2_O_2_ presoaking prior to AFEX is performed that maximizes the lignin removal at 37.1% with 0.5 (g/g of dry biomass) H_2_O_2_ loading. Ultimately, this is due to more degradation of polysaccharide, forming more lignin droplet which get re-condensed to form new macromolecular lignin as pseudo lignin that might be formed from the degradation of hemicellulosic content [101]. Another variation is the CO_2_-loaded ammonia explosion pretreatment method in which CO_2_ under high pressure penetrates the biomass and results in the increase in pore size of lignocellulosic biomass which is far better than using ammonia explosion which only loosens the bond between lignocellulosic biomass and increases the glucan conversion up to 90% [104]. An alternative form of ethanol production occurs by using aqueous ammonia at 27% (w/w) at 27 ℃ for 14 days. This causes cleavage of the C–O–C bond between lignocellulosic biomass with a fermentation efficiency of 96% of the total ethanol yield [105]. Similarly, ammonia carbonate (AC) solution can be used as the substitute for ammonia pretreatment as AC is considered a weak base and one of the promising solvents for pretreatment, as it acts as a CO_2_ capture system. It is also observed that AC increases the external surface area of rice straw and eliminates the amorphous fraction available in the biomass and is intended to increase the enzymatic digestibility for further process. It has been observed that AC at 25% (v/v) for 12 h improves enzymatic saccharification of up to 67.7%, glucan content recovery to 40.9%, and bioethanol yield up to 10.61 g/l, i.e., 47.78%. The lignin–hemicellulose bond is successfully attacked by AC and is particularly good in cleaving ester and ether bonds in lignin–carbohydrate structures and is also excellent at cleaving the ester and C–C bonds in lignin molecules, increasing the digestibility of cellulose [106]. Ammonium pretreatment with its different substitutes ultimately enhanced the yield and concentration of ethanol obtained. Yet in another method, it was reported that by combining CO_2_ with the AFEX process at varying temperature conditions ranging from 25 to 250 ℃ for 1000–4000 psi pressure, a maximum yield of 93.6% was obtained at 165.1 ℃ temperature for 69.8 min residence time at 2.2 MPa of CO_2_ loading, along with 14% of ammonia concentration. This process removed lignin effectively along with induction of swelling of lignocellulosic material while CO_2_ pierced into the feedstock at high pressure, which led to the expansion of the surface area of lignocellulosic biomass (LCBs) and residual CO_2_ could be collected and recycled for various other usages [104].

AFEX pretreatment is almost similar to that of the steam explosion method, where LCB is treated with ammonia at a specific temperature (600–1000 ℃) and high pressure of around 250–300 psi for a short period. This includes both physical (temperature and pressure) and chemical (ammonia) parameters to decrease the lignocellulosic recalcitrant structure for hydrolysis [62]. Through AFEX process structural modification in the physical and chemical properties takes place in the plant cell wall. The AFEX is exposed at a pilot scale with maximum ammonia revival and is considered an effective pretreatment for LCBs through cleavage of the ester-linked lignin–carbohydrates complex that effectively damages the rice straw structure. The advantages of using ammonia pretreatment are its usage at the industrial level, with the toxicity of the inhibitor remaining low, and no water washing is required hence AFEX is a simplified process.

### CO_2_ explosion

CO_2_ explosion pretreatment is associated with a mechanism to that of steam explosion pretreatment. In the execution of the process, we feed CO_2_ within high-pressure barrel-bearing biomass accompanied by agitation at the requisite temperature and administering for a desirable period at approximately 200 ℃ temperature. In this process, carbonic acid formed by CO_2_ diffuses into the biomass that can be used to hydrolyze hemicelluloses. After the hemicellulose hydrolyzation, pressure is subsequently increased, which ultimately disrupts the biomass structure, this results in increased accessibility of cellulose and is considered a green solvent with non-toxic properties [107]. Earlier, it was reported that CO_2_ explosion assisted with AFEX resulted in 93.6% of glucose yield. Furthermore, high-pressure CO_2_ explosion led to 12.4% of hemicellulose content removal from the peanut shells. This facilitates the consequent transformation of glucose by saccharification with the highest glucose yield of 80.7% at 190 ℃ along with 60 bar CO_2_ pressure [108]. It is estimated that high-pressure CO_2_ has increased the pore size between cellulose and hemicellulose thus leading to a consequential increase in the porosity of biomass after pretreatment and improvement in the enzyme adsorption capacity. The pressurized gas disrupts the biomass structure which ultimately increases the accessibility of the exterior part of biomass for further processing [109].

The advantage and suitability over other pretreatment processes are that it is comparatively cheaper with a higher yield of reducing sugar and requires relatively low temperature. However, this process includes the use of a costlier reactor that should be able to withstand the high pressure of CO_2_ explosion. The selection of biomass is an important parameter where low moisture content biomass is the main obstacle for performing this pretreatment.

### Wet air oxidation

Wet air oxidation (WAO) involves the oxidation of organic and oxidizable inorganic components at a temperature nearly about 170–200 ℃ for a short reactor time, i.e., 5–15 min, and high pressure around 10–20 MPa performed on rape straw biomass. In this process, high O_2_ concentration acts as the sole chemical and catalyst utilized as an additional oxidative agent. The pretreatment process takes place with the formation of hydroxyl radicals and autocatalyzes further by forming organic acid [110]. The optimum parameters involved in pretreatment are reaction temperature, oxygen pressure, time for presoaking of biomass and recycling of substrate but a high cost is required for performing the WAO. Analysis of various pretreatment methods including steam explosion, hydrothermal process and wet air oxidation are carried out comparably, concluding that wet air oxidation at 200 ℃ resulted in higher glucan concentration while minimal xylan degradation due to the thermal effect on *Chaetomorpha linum* biomass. This was most likely brought on by the decomposition of sugar monomers, which prevented the extraction of carbohydrates [111]. Furthermore, the optimized condition for WAO process with 89% removal of lignin along with 69.77% solubilization of hemicellulose was performed at 185 ℃ temperature, with 0.5 MPa pressure for 15 min performed on rice husk. At a more elevated temperature (195 °C), when coupled with rising pressure and reaction time, decreased cellulose recovery was seen, indicating unfavourable cellulose breakdown. Hemicellulose concentration in the solid fraction was minimal because the majority of lignocellulosic materials had been solubilized under the WAO conditions and resulting in the slurry's dark brown colour [112]. Some of the reported, improved WAO processes require the involvement of alkaline peroxide assistance with WAO through hydrolysis, using 72% H_2_SO_4_ at 30 ℃ and is considered a two-step pretreatment process, enhancing the enzymatic digestibility along with improvement in cellulosic content. This was similar to the one of composite material, with silica serving as the matrix and cellulose, hemicellulose, and lignin serving as the "cementing" components [113]. Further increasing the temperature for wet air oxidation to 210 ℃ for 3 min at approximately 12 bar of oxygen results in 86% revival of cellulose and hemicellulose from the lignin counterpart with 67% of ethanol output from rape straw [110]. The ideal condition for the wet air oxidation process on ryegrass with a maximum glucose yield of 75.5% along with cellulose recovery of 95.2% was obtained at 195 ℃ for an incubation time of 10 min and oxidation at 1.2 MPa with a solid-to-liquid ratio of 1:15 g/g. It was evaluated that poor convertibility obtained in the pretreatments with WAO at temperatures below 185 °C was ineffective for enhancing the enzymatic saccharification of clover–ryegrass biomass [114].

## Biological pretreatment processes

The aforementioned biological pretreatment method is preferred over other pretreatment methods due to its low energy requirement, eco-friendly process with less generation of pollutants as no chemical is required and simpler one with a broad assemblage of taxonomical microorganisms available naturally for the process [115]. These microorganisms are selected as bacteria, fungi or actinomycetes depending upon the substrate. Biological pretreatment involves various white, brown or red rot fungus-secreting cellulolytic enzyme that breaks the bond between cellulose and lignin, increases the accessibility of cellulose, increases porosity, and lets cellulose be available for further hydrolysis and fermentation process [116]. Within this microflora, a variety of microorganisms are available such as *Clostridium* *sp*., *Cellulomonas* sp., *Bacillus* sp., *Thermomonospora* sp., *Streptomyces* sp., *Phanerochaete* c*hrysosporium*, *Trichoderma reesei,* *Trichoderma viride, Aspergillus niger,* etc. [117]. Furthermore, white-rot fungi such as *Pleurotus sp.* and *Trametes sp*. are outlined for their high titers of ligninolytic and hydrolytic enzyme potency or through their lignin degradation enzymes such as peroxidase and laccase [118].

Biological pretreatment is believed as an eco-friendly solution over the thermochemical process. On comparing, biological pretreatment with the steam explosion process, it can be concluded that after pretreatment, cellulose content in steam-treated biomass is 39.5% while in biological pretreatment is 37.6%, whereas lignin removal is higher in biological pretreatment as compared to steam exploded pretreatment [119]. The use of biological therapies, which rely on microbial processes to hydrolyze the accessible polysaccharides and destroy lignin, appears to be environmentally friendly because it uses lower energy and hardly any chemicals. But the lengthy residency restrictions must be addressed while further processing biological pretreatment. In addition, combining biological pretreatment with alkali has improved the efficiency of pretreatment by decreasing the reaction time with less concentration of alkali. For example, oxidative pretreatment along with *Pleurotus eryngii* increases the reducing sugar yield from 1.10 to 1.29 folds in hemp woody core in 21 days and the glucose yield up to 271.1 mg/g, hemp chips' lignin level can be reduced by bio-pretreatment, which also makes it easier for hydrolytic enzymes to reach the highly ordered cellulose crystal structure. [118].

Lignocellulosic biomass (LCBs) comprises cellulose and hemicellulose hydrolyzed by their respective cellulolytic and hemicellulolytic microorganism under aerobic and anaerobic conditions. Cellulolytic microorganisms are *Clostridium sp., Thermanaerovibrio acidaminovorans, Thermoanaerobacterium thermosaccharolyticum, Beta proteobacterium, C. clariflavum, Paenibacillus* [120]*.* These microorganisms secrete cellulose-binding proteins that produce endoglucanase and xylanase, which degrade the lignocellulosic structure of the biomass. Similarly, the crust fungi, *Phanerochaete chrysosporium* assisted with acid pretreatment using 1% H_2_SO_4_ for 60 min at 100 ℃, is well known for the breakage of β-glycosidic bond in lignin that leads to the biological release of reducing sugar at around 370 mg/g from 400 µm particle size biomass [121]. This fungus degrades lignin by producing various enzymes, such as laccase, manganese peroxidase and lignin peroxidase, that are required for the process of bioconversion [122]. Enzyme production from biological pretreatment is a slow process and requires a large part of carbohydrates for its formation. To overcome such a problem, employment of biological pretreatment with various microbial consortia can be carried out using specific reactors that reduce the pretreatment time and overcomes the loss of carbohydrate during the process [123]. Biological pretreatment of feedstock and the compositional analysis of cellulose (a polymer of β-D- glucose), hemicellulose (polymeric carbohydrate of xylan, arabinose and glucomannan) and lignin (polyphenolic complex structure) is determined using various microorganisms that optimize the production of enzyme for the degradation of lignocellulosic biomass (LCB) structure. This process is considered a relatively gradual and time-consuming process, but the yield obtained from the particular strain of microorganism, which may be bacterial or fungal is ameliorated due to the effective delignification of the biomass, The advantages and disadvantages of the process are summarized in Table 3.

**Table 3:** Advantages and disadvantages of using biological pretreatment processes [119, 122, 138]

Advantages	Disadvantages
Low capital investment as the bacteria and fungi are easily extracted from natural sources	Long microbial pretreatment time generally requires 15–20 days for maximum delignification of biomass
Low energy requirement as it is incubated at 32 ℃ temperature hardly for 72 h	Lower content of soluble hydrolysate is obtained with an efficiency rate of 37–40%
Efficient and environmentally safe as microbes used during the process are not hazardous as in the case of chemical and physiochemical pretreatment	Tardy rate of saccharification of LCBs as 30% lignin removal has occurred during bacterial action

### Fungal pretreatment

Biological pretreatment using various micro-organisms such as brown, white and red rot fungi. These have lignin degradation aptness while brown fungi have the intention to degrade cellulose and hemicellulose with little degradation of lignin content. Similarly, white rot fungi have been more effective in degrading cellulose, hemicellulose and lignin at the same rate [119]. The high efficiency of lignin degradation by white rot fungus is facilitated by laccase, polyphenol oxidase and manganese peroxidase enzyme formation [124]. The fungal pretreatment efficiency potency mainly depends on the fungal growth and its metabolism process in the form of biomass particle size, incubation time as well as moisture content [122]. Appropriate moisture content is required for lignolytic activities in the biomass. On comparing pretreatment efficiency, strain *P. ostreatus* shows a lignin removal capacity of 33.4% while *T. reesei* exhibits lower lignin removal of 23.6% in 30 days of incubation time [125]. Various fungi used for lignin degradation are *Ceriporiopsis subvermispora*, *Phellimus pini*, *Phlebia spp*., *Pleurotus spp*., and *Trametes hirsute*. *Myrothecium roridum* shows higher glucan recovery of up to 70% [124]. Another white rot fungus *Irpex lacteus* of class basidiomycetes shows a maximum lignin reduction of 41.5% in the case of corn stover along with the highest xylan digestibility of about 82.1% and the highest hydrolysis result is obtained after 15 days of fungal pretreatment [126]. One more fungus *Trichoderma harzianum* is used as a nitrogen source in the biomass pretreatment, cultured in potato dextrose media and is capable of producing cellulase, xylanase and glucosidase for hydrolysis process with maximum sugar yield and enhancing the fermentation process in 96–120 h of reaction time [127]. It is reported that *Phanerochaete chrysosporium* shows maximum lignolytic activity due to the secretion of peroxidase enzyme. The microbial pretreatment assisted with 4% NaOH or 1% H_2_SO_4_ resulted in maximum cellulosic content up to 39.4% after soaking at 100 ℃ for 1 h. Thus, it is demonstrated that numerous fungi have been shown to increase the digestibility of polysaccharides, but only a small number of species have been able to degrade significant amounts of lignin while preserving cellulose and some hemicellulose [128]. Similar fungal pretreatment using *Fusarium oxysporum* considered a filamentous fungus has high zinc metal tolerance capability and produces a variety of enzymes. Thus, zinc oxide nanoparticle is used to enhance the production of bioethanol as it is used as a reducing, capping and stabilizing agent during fung*al* pretreatment. During the combined process, lignin degradation occurs from 25% to 16.7% while cellulose recovery reaches up to 48.8% [129].

### Bacterial pretreatment

Biological pretreatment assisted with bacterial treatment showed higher lignin degradation than fungal pretreatment due to its easier genetic manipulation and its tolerance towards environmental conditions. Various bacteria such as *Sphingobium* sp. SYK-6, *Rhodococcus* sp.*, Ceriporiopsis* sp., *Pandoraea* sp., *galactomyces* sp., and *Mycobacterium* sp. used for bacterial pretreatment show efficient lignin and polycyclic aromatic hydrocarbon degradation prior to cellulose. The bacterial strain *Mycobacterium smegmatis* L2-K2 grows in the medium containing glucose as the carbon source required during the process [130]. These bacteria have engulfed the cell wall of biomass and released various enzymes such as cellulase, xylanase, amylase, and several other lignolytic enzymes required for the subsequent process of bioethanol production [131]. *Bacillus firmus* is considered a xylanolytic enzyme-producing bacteria used to pre-treat the biomass that has enhanced glucan recovery yield by up to 74% and increases xylan removal to 30%, this subsequently increases the availability of cellulose towards its respective enzymes [132]. *Galactomyces* sp. produces cellulase enzyme up to 15 mg/g of glucan along with xylanase and β-glucosidase. This has increased the reducing sugars yield of up to 81% while glucose up to 74.1% from 100 gm of biomass. The cocktail of biological species has increased the successive yield from the hydrolysis and fermentation process [133]. The bacterial strain of *Pandoraea sp.* produces manganese peroxidase, a laccase that disintegrates the recalcitrant structure of lignocellulosic biomass along with the removal of lignin and hemicellulose from the biomass. A wide variety of tiny molecules that act as diffusible mediators and directly react with lignin to produce both phenyl and phenoxyl radicals on the biomass are involved in the oxidation of lignin. These radicals then set off a series of bond scission events. Bacteria may react differently to lignin than fungi do; in other words, the bacterial system could not be entirely controlled by small-molecule oxidants [134]. Another bacterial strain used for biological pretreatment includes *Streptomyces griseorubens* whose degradative activities depend on various factors such as residence time, pH, temperature and enzyme loading. With an increase in the incubation time, an increased impact on the delignification rate of rice straw biomass was observed with an increase in the amount of accessible cellulose up to 64.77% and a consequent reduction in the contents of hemicellulose and lignin up to 86.13% and 93.31%, respectively [135]. A bacterial strain sourced from termites’ gut is *Ochrobactrum oryzae* which can enhance the hydrolysis output through its degradative action. After 16 days of pretreatment, cellulosic content enhances up to 22.38%, hemicellulose to 18.64% while lignin removal reaches 44.47% from the biomass with conversion efficiency of 51.92%. During the bio-delignification process, the bacterial strain keeps cellulose and hemicellulose intact. This ability increases the quantity of reducing sugars that are accessible, which improves biofuel output [136]. Thus, this lignolytic bacteria has a superior metabolic process and exhibits a greater growth rate as compared to fungal pretreatment. Due to its higher metabolic rates, it requires lesser time for the pretreatment process, which is 3 to 11 days in presence of aerobic conditions [137]. In the bacterial system, there are several direct interactions between LCB and enzyme which could be the main cause of the bacterial system's poor performance in the pretreatment described here. Therefore, the presence and dispersion of mediators may be necessary conditions for the progression of lignin degradation during biological pretreatment [134].

Due to the longer residence time, biological pretreatment is combined with various other methods that decrease the drawbacks of biological pretreatment and increases the overall outcome of the process [138]. The microorganism used for both bacterial and fungal pretreatment are isolated from various sources and obtained enzymes are assessed and optimized to obtain various reducing sugar. Table 4 illustrates the microbial strains obtained from both fungal and bacterial pretreatment along with the enzyme produced from that particular strain and the composition of sugar after biological pretreatment.

**Table 4:** Biological pretreatment using various microalgal strains and its effect after pretreatment on biomasses

Microbial strain	Isolated from	Enzyme produced	After pretreatment	References
Fungal pretreatment				
* Pleurotus florida*	Cultured on potato dextrose agar media	Cellulase	19.9% delignification, 83.2% cellulose recovery	[116]
* Pleurotus eryngii*	Cultured on potato dextrose plate, 28 ℃ for 7 days	Xylanase, laccase	42.4% lignin degradation, 85.6% cellulose recovery	[118]
* (Phanerochaete chrysosporium*	Potato dextrose agar media, 28 ℃, 84 h	Arabinose, xylanase	41.23% cellulose, 29.34% hemicellulose, 3.66% lignin	[121]
* Trichoderma reesei *& *Pleurotus ostreatus*	Cultured on potato dextrose agar media		33.4% lignin removal	[125]
* Irpex lacteus*	Obtained from mycelium culture	Cellulase	59.2% glucan digestibility, 45.8% lignin degradation	[126]
* Trichoderma harzianum*	Cultured on potato dextrose agar media	Cellulase, β glucosidase, xylanase	Increase ethanol production by up to 38.2%	[127]
*Fusarium oxysporum* (Zinc oxide nanoparticles)	Potato dextrose agar media, 121 ℃, 20 min	Cellulase, xylanase, β-glucosidase	48.8% cellulose, 16.7% lignin	[129]
Bacterial pretreatment				
* Galactomyces* sp*.*	Inoculated in growth media containing 1 g (NH_4_)_2_SO_4_, 0.05 g K_2_HPO_4_, 0.05 g MgSO_4_.7H_2_O, 0.05 g CaCl_2_, at 180 rpm for 72 h	Cellulase, β-glucosidase, xylanase	50.6% cellulose, 7.4% hemicellulose, 12.6% lignin	[133]
* Pandoraea* sp.	Bamboo	Manganese peroxidase and laccase	42.6% cellulose, 41.6% hemicellulose, 12.9% lignin	[134]
* Streptomyces griseorubens*	The microbiological culture collection centre	Cellulase and xylanase	After 20 days, 65.63% cellulose, 11.89% hemicellulose, 2.81% lignin	[135]
* Ochrobactrum oryzae*	Termite gut	Xylanase	52% cellulose, 24.05% hemicellulose, 10.04% lignin	[136]

## Integrated pretreatment of various feedstock

Individual pretreatment approaches for various feedstocks yield incremented output for final-level bioethanol production. The steps of these pretreatment approaches if integrated into combined approaches can further substantiate the output for a final product along with the reduction in time and chemicals and could qualify for being green energy processes. Innovative combinations of two or more pretreatment procedures are currently of particular interest for efficient digestibility and selective biomass retrieval. The combination of extrusion process with ultrasonic effect is considered a chemical-free and eco-friendly pretreatment technique performed on rice hull biomass with a 77.5% of conversion rate. With the utilization of ultrasonic wave that produces extremely high temperature and pressure along with strong shear force disrupted the crystalline state of cellulose and subsequently improved further hydrolysis yield [24]. Application of combined steam explosion technique with choline chloride resulted in efficient expulsion of 84.7% lignin with 4.5 times higher glucose yield than that of a single pretreatment process. In this choline chloride is used as a deep eutectic solvent, acting as Lewis acid when dissolved in water, and has the capability of revising the crystallinity of cellulose as it becomes viscous at the higher temperature, thus requiring longer pretreatment time. To overcome this, issue steam explosion at 1 MPa and 184 °C temperature with ChCl pretreatment is performed on corn stover biomass [139]. Similarly, incorporating H_2_O_2_ with a steam explosion is considered an environmentally friendly and secure method for producing bioethanol that is both an economical and sustainable approach. Due to the existence of the extremely reactive hydroxyl radical (OH^−^) and superoxide anion radical (O_2_^−^), H_2_O_2_ is employed for delignification. Through oxidation and breakdown, these active radicals delignify the structure of lignocellulosic biomass and improve the further processing of biomass with hydrogen peroxide combined with citric acid [140].

The inclusion of chemicals during ball milling can significantly improve specific interactions between biomass and catalyst that result in the amplification of the process. Furthermore, the high energy generated by ball milling enhances the overall catalytic process. Even though mechano-catalytic pretreatment of biomass has previously been investigated in terms of pretreatment temperature, time, and chemical concentration. The presence of a small quantity of dilute alkali during ball milling can effectively facilitate lignin dissolution in the subsequent hydrothermal pretreatment process [33]. It was estimated that high xylose yield using hot compressed water treatment (HCWT) under mild conditions had the potential to dissolve xylose and arabinose of the hemicellulosic part of rice straw biomass. Combining mild HCWT with wet disk milling (WDM) is considered an environment-friendly pretreatment technique with maximum sugar yield obtained after enzymatic hydrolysis [34]. Alkaline pretreatment is considered economically unfeasible as it takes a longer time to complete and requires more alkali for biomass pretreatment, but physical methods such as ultrasound can be used in addition to alkaline pretreatment to speed up the process [40]. Similarly, combining twin screw extrusion with an alkali using 0.6 M NaOH, results in the formation of black Liquor, containing phenolic content from lignin counterpart and considered hazardous to aquatic flora and fauna. Thus, recycling black liquor for further processing, subsequently reduced the cost involved in the production of bioethanol. The recycled black liquor after adjusting the concentration of NaOH was further used as the catalyst for further combined process of the screw extrusion method [36]. Another combined pretreatment technique is the involvement of hydroxymethyl reagent along with alkaline pretreatment, as it enhances the hydrophilicity of lignin by attaching the hydroxymethyl group at ortho position of the aromatic position. The non-productive adsorption of enzyme and lignin is thought to be caused by hydrophobic interaction at the ortho position. Thus, it is believed that a pretreatment involving lignin content reduction using NaOH treatment and lignin structure modification using hydroxymethyl reagent treatment, are devised to reduce the adverse effects of lignin on enzymatic hydrolysis [51]. High-pressure CO_2_ at 60 bar pressure during hot water treatment has increased the porosity of biomass as well as enhance the capacity of enzyme adsorption on cellulose. During the process, CO_2_ forms carbonic acid that decomposes hemicellulose effectively and makes cellulose easily available for conversion to glucose by subsequent enzymatic activity. The enhanced acidic concentration during carbonic acid concentration in water significantly decreases the hemicellulosic content of peanut shell biomass used for bioethanol production [109]. The particle size has a greater effect in deciding the effect of pretreatment on biomass. The smaller particle size exhibits easily available reactive site for further processing as well as enhancing the pretreatment time. It is noted that smaller particle size tends to release more amount of reducing sugar after the saccharification process. Using combined pretreatment of grinded biomass with particle size 400 µm and further microbial treatment with *Phanerochaete chrysosporium* along with chemical treatment using 1% H_2_SO_4_ yielded 370.23 mg/g of reducing sugar, manifested by the exposure of maximum surface area of biomass to microbial cells as well as the acidic reaction that enhances the pretreatment reactions [121].

Hydrogen peroxide soaking prior to AFEX enhances the pretreatment reaction, as H_2_O_2_ is considered a strong oxidant with environment-friendly properties and an inexpensive solvent used in combination with AFEX. The AFEX-pretreated sample shows less water washing after treatment and efficient structural modification of biomass and could thus be considered a simplified process among various pretreatment techniques [101]. With all this assessment, it becomes confirmed that low H_2_O_2_ loading during AFEX showed maximum delignification and maximum retention of polysaccharides during processing. During CO_2_-assisted AFEX pretreatment, CO_2_ penetrates deep into the biomass causing increment in the pore size of biomass proliferating ammonia bringing swelling in the biomass that leads to the removal of lignin efficiently [104]. Another advanced pretreatment technique involves 2-methyl tetrahydrofuran as a biomass-derived solvent with a low boiling point of 80 °C which is considered an environmentally friendly source and can easily be recycled for further use. This solvent system can be combined with microwave-assisted degradation which further increases the enzymatic activity performed on pretreated biomass and causes accelerated hemicellulose removal and exposes the cellulose for enzymatic action [99]. Similarly, microwave radiation is combined with dilute acids and high-pressure treatment in which cellulose is partially broken down with raised temperature, while hemicellulosic structure breakdown occurs by treatment of weak acid and lignin which is subjected to high temperature and pressure leading to the breakdown of recalcitrant structure [38].

One of the most extensive pretreatment methods is a steam explosion, which expands the convenient lignocellulosic plant cell walls, induces hemicellulose deterioration and lignin transition, and thus enhances the potential for cellulose hydrolysis. To increase the efficiency of hemicellulose hydrolysis, acid-impregnated steam explosion technique is performed to obtain hydrolysate rich in xylose content. It is estimated that acid-impregnated steam explosion shows 85.3% of cellulose conversion which is 1.6 times more than that of acid pretreatment using corncob biomass [88]. By primarily removing hemicellulose and modifying cellulose properties, steam explosion (SE) has been used as a reasonably inexpensive and environmentally acceptable technique. Thus, combining steam explosion with green liquor pretreatment is thought to be a mild alkaline delignification method that selectively removes lignin while retaining as much cellulose and hemicellulose as possible in biomass residues, resulting in less hazardous or corrosive byproducts [89]. Pretreatment using liquid hot water necessitates high temperature, less lignin removal rate, and high enzyme loading. As a result, a suitable mix of pretreatment technologies should not just improve lignocellulosic biomass digestibility at a low operational cost, but also enhance lignocellulosic component usage. Hence, LHW along with alkaline pretreatment NaOH/O_2_ [96] and Na_2_CO_3_/O_2_ [93] has enhanced the rate of xylan recovery and lignin removal as well as increased digestibility of enzyme during hydrolysis. As elaborated above various combined pretreatment process effectively enhances carbohydrate recovery from its persisting complementary compounds. This helps in promoting the enzymatic digestibility of lignocellulosic biomass through the process of lignin removal. The effect of combined pretreatment on the sugar composition, both before and after the pretreatment process along with the impact of conditions such as temperature, pH and concentration of various chemicals required during the process is illustrated in Table 5 [141].

**Table 5:** Various integrated pretreatment methods and their outcome

Integrated pretreatment methods	Biomass	Compositional analysis of biomass		Pretreatment conditions	Reference
		Before pretreatment	After pretreatment		
Microwave + acid + alkali pretreatment	Rice straw	42.54% cellulose, 24.51% hemicelluloses, 9.16% lignin	60.07% cellulose, 14.90% hemicellulose, 4.52% lignin	1% NaOH 1% H_2_SO_4_ 2450 MHz frequency, 850 W and 150 ℃ for 3 min	[15]
Two-stage liquid Hot water with alkali oxygen pretreatment	Wheat straw	46.65% cellulose, 20.26% hemicellulose, 29.05% lignin	63.98% cellulose, 18.28% hemicellulose, 16.37% lignin	18% (w/w) Na_2_CO_3_ oxygen pressure 689 kPa, 12 h, 60 ℃, LHW^a^ 216 ℃ for 10 min	[97]
Extrusion + ultrasound	Rice hull	35.56% cellulose, 13.68% hemicellulose, 19.21% lignin	77.5% conversion rate of cellulose and hemicellulose	60 mesh, temperature 143 ℃; speed of screw at 350 rpm	[24]
Ball milling + alkali pretreatment	Sugarcane bagasse	37.3% cellulose, 16.6% xylan and 24.9% lignin	62.5% cellulose, 18.8% hemicellulose and 18.4% lignin	100 ℃ for 50 min 4% NaOH	[33]
Hot water treatment + wet disk milling (mechanical process)	Rice straw	31.2% glucose, 12.9% xylose, 2.6% arabinose	94% glucose, 84%xylose, 69% arabinose	135 ℃ for 60 min 9000 rpm for 10 min	[34]
Alkaline twin-screw extrusion pretreatment	*Miscanthus sacchariflorus* biomass	40.3% cellulose, 24.1% hemicellulose, 24.1% lignin	58.9% cellulose, 34.1% hemicellulose, 12.6% lignin	100 ℃, 0.6 M NaOH screw speed of 80 rpm for 8 min	[36]
Microwave-assisted dilute Acid	Wheat and rye stillage	16.80% cellulose, 29.62% hemicellulose, 15.57% lignin	29.67% cellulose, 28.16% hemicellulose, 9.72% lignin	0.2 M H_2_SO_4_ applied power at around 300 W, 15 min, 372 kPa	[38]
Ultrasonic + alkaline pretreatment	Rice straw	52.38% cellulose, 14.98% hemicellulose, 23.69 lignin	71.27% cellulose, 7.95% hemicellulose, 19.61% lignin	30 min ultrasonication 0.5% (w/v) NaOH	[40]
Sonification assisted with dilute Acid pretreatment	Rice straw	36.5% glucan, 20.8% xylan, 16.9% lignin	Total sugar yield 31.78 g/ 100 g of biomass	Sonification at 80 ℃ for 50 min, 10% (v/v) H_2_SO_4_	[42]
Alkali pretreatment and hydroxymethyl reagent	Sugarcane bagasse	46.88% glucan, 22.73% xylan, 22.52% lignin	61.46% glucan, 24.10% xylan, 12.38% lignin	1 M NaOH, 1.5 M formaldehyde, 80 ℃ for 2 h	[51]
Alkali salt soaking pretreatment	Sugarcane bagasse	38.11% glucan, 22.61% xylan, 16.67% lignin	36.2% glucan, 21.48% xylan, 6.45% lignin	Alkali salt- 0.5% K_2_CO_3_ and 0.068% K_2_SO_3_ at 120 ℃ for 40 min	[55]
Alkali pretreatment assisted with high pressure	Cotton stalk	39.85% cellulose, 24% hemicellulose, 23.92% lignin	64.07% cellulose	3% NaOH, 130 kPa pressure for 40 min	[56]
Phosphoric acid and hydrogen peroxide (PHP) pretreatment	Wheat straw	33.3% cellulose, 19.8% hemicellulose, 21.7% lignin	87.12% cellulose recovered, 30.1% lignin removal	74.92 g H_3_PO_4_ (85% w/w), 5.08 g H_2_O_2_ (30% w/w), 180 rpm, 40.2 ℃ for 2.9 h	[60]
Microwave-assisted with CaCl_2_/CrO_3_/NiCl_2_ + H_3_PO_4_	Rice straw	24.9% cellulose, 30.9% hemicellulose	CaCl_2_ + H_3_PO_4_- 12.4% cellulose, 14.77% hemicellulose, CrO_3_ + H_3_PO_4_-30.5% cellulose, 21.7% hemicellulose, NiCl_2_ + H_3_PO_4_ 39.2% cellulose, 27.4% hemicellulose	0.25 M CaCl_2_/NiCl_2_/CrO_3_ and 3% (v/v) H_3_PO_4_, 700 W magnetron, 155 ℃ temperature for 20 min	[65]
Microwave-assisted ionic liquid Pretreatment	Rice straw	34.32% cellulose, 17.87% hemicellulose, 20% lignin	35.58% cellulose, 17.09% hemicellulose, 15.86% lignin	20 gm [BMIM]Cl^b^ 130 ℃ for 45 min 400 W power	[67]
PEG-assisted ionic liquid pretreatment	Sugarcane bagasse	42.3% glycan, 22.7% xylan, 22.7% lignin	65.3% glycan, 27.8% xylan, 3.3% lignin	[BMIM]Cl + 5% polyethylene glycol 154.6 °C, 60 min	[74]
Deep eutectic solvent assisted with ultrasonic wave	Sugarcane bagasse	38.6% cellulose, 20% hemicellulose, 24.68% lignin	42.7% cellulose, 27.86% hemicellulose, 5.4% lignin	1 mol of ChCl^c^, 10 mol glycerol, ultrasonic wave of frequency 20 kHz for 20 min	[82]
Liquid Hot Water + sodium carbonate-oxygen pretreatment	Reed	38.7% glucan, 21.8% xylan, and 24.4% lignin	80.4% glucan, 16.3% lignin	Oxygen pressure 0.8 MPa, 16% N_2_CO_3_, 160 ℃ for 60 min	[93]
High-pressure CO_2_ hydrothermal pretreatment	Peanut shell	35.7% cellulose, 20.6% hemicellulose, 34.1% lignin	38.8–45.3% cellulose, 12.4% hemicellulose, 40.8% lignin	190 ℃ for 1 h at 60 bar CO_2_	[109]
CO_2_ + ammonia explosion pretreatment	Rice straw	31.8% cellulose, 17.5% hemicellulose, 18.2% lignin	41.5–57.6% cellulose, 17.2–23.7% hemicellulose, 7.3–15.5% lignin	Ammonia concentration at 14.3%, 2.2 MPa CO_2_, 165.1 °C for 69.8 min	[104]
Acid-impregnated steam explosion pretreatment	Corn cob	41.5% cellulose, 35.8% hemicellulose, 16.2% lignin	64.4% cellulose, 5.2% hemicellulose, 26.5% lignin	2% H_2_SO_4_ 100 ℃, 120 min	[88]
Steam explosion + green liquor solution (Na_2_S and Na_2_CO_3_	Bamboo	38.29% cellulose, 28.50% hemicellulose, 28.41% lignin	62.44% cellulose, 13.38% hemicellulose, 20.08% lignin	Steam explosion at 213.3 ℃ for 5 min, green liquor 31% (w/w), 166.41 ℃ for 28.01 min	[89]
Steam explosion assisted with potassium hydroxide (SEKOH)	Rice straw	37.4% cellulose, 24.46% hemicellulose, 28.8% lignin	65.6% cellulose, 4.58% hemicellulose, 8.5% lignin	Steam explosion with the applied temperature at 170 °C for 10 min with 2% (w/v) KOH and autoclave at 121 °C for 30 min	[92]
Liq. Hot water + sodium hydroxide/oxygen pretreatment	Reed	39.54% glucan, 22.23% xylan, and 21.03% lignin	34% glucan, 5.46% xylan, and 11.3% lignin	Oxygen pressure 0.6 MPa, 8% (w/w) NaOH, 160 ℃ for 60 min	[96]
Biphasic 2-methyl tetrahydrofuran (MeTHF) + oxalic acid + water pretreatment	Bamboo	46.88% cellulose, 26.73% hemicellulose	72.17% cellulose, 0.44% hemicellulose	1 mol/L oxalic acid as catalyst + 2-MeTHF^d^ frequency 2500 MHz 180 ℃ for 20 min	[99]
AFEX^e^ + hydrogen peroxide presoaking	Energy crops (bamboo, giant reed, and Miscanthus	66.2% glucose, 26.2% xylose,	76% glucose, 12.7% xylose	30% H_2_O_2_, 2.0 ammonia loading, 130 ℃ for 10 min	[101]
Milling, along with microbial treatment followed by acidic pretreatment	Sesame plant residue	22.80% cellulose, 37.76% hemicellulose, 7.35% lignin	41.23% cellulose, 19.34% hemicellulose, 3.66% lignin	400 µm biomass size, *Phanerochaete chrysosporium*, 1% H_2_SO_4_	[121]
Steam explosion + Choline chloride (ChCl)	Corn Stover	46% cellulose, 19.26% hemicellulose, 20.31% lignin and 2.51% ash	74.59% glucan, 7.51% xylan, 6.22% lignin	1 MPa, 184 °C for 15 min Biomass and ChCl ratio 1:2.2 (w/w)	[139]

^a^Liquid hot water

^b^1- Butyl-3-methylimidazolium chloride

^c^Choline chloride

^d^2-Methyl tetrahydrofuran

^e^Ammonia Fibre Explosion

**Fig. 6:**
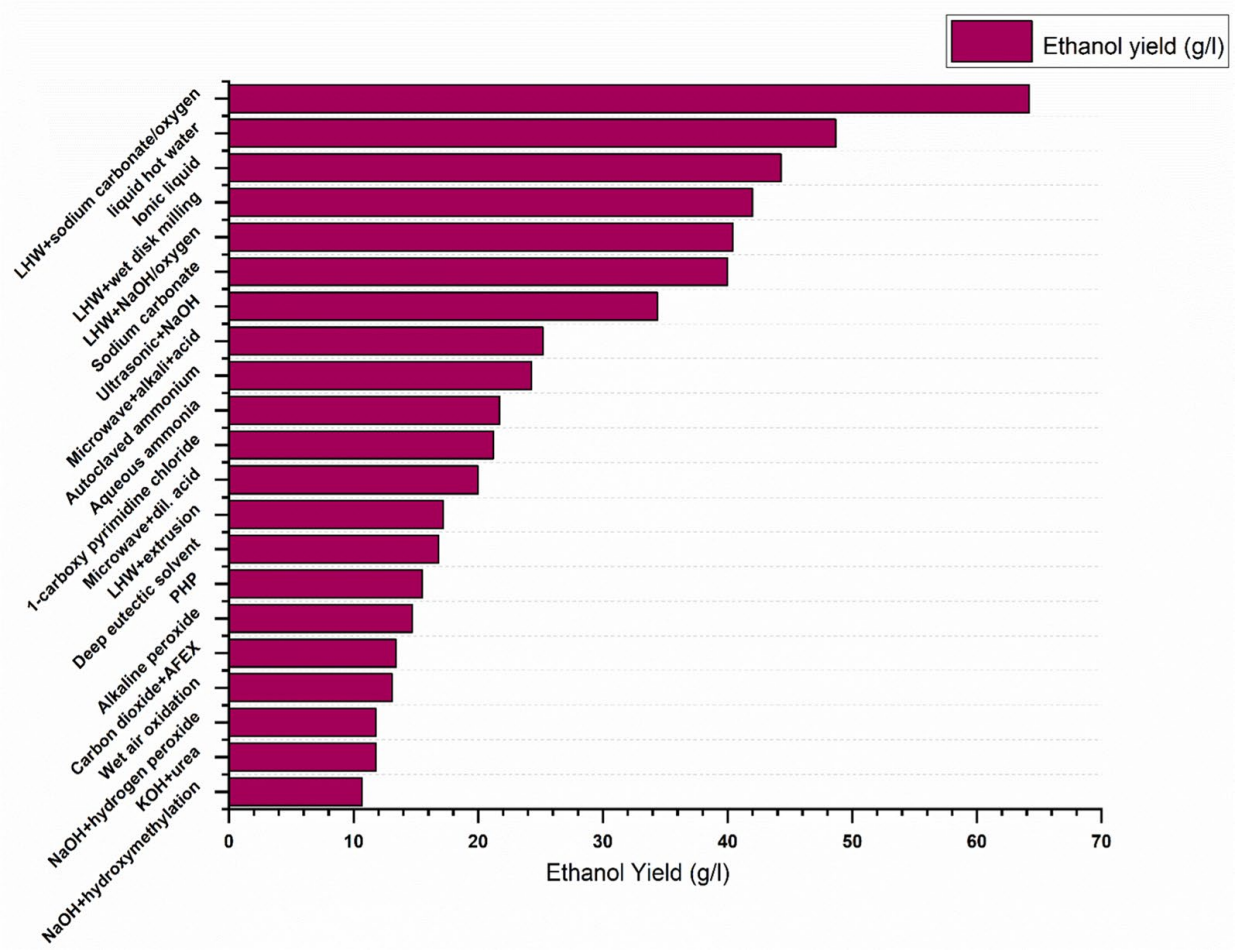
Demonstration of the ethanol yield obtained from various integrated pretreatment technique after further processing steps

During chemical pretreatment, lignin is fractionated into the following groups: solid lignin, soluble lignin, phenolic derivatives, carboxylic acids, CO_2_ and H_2_O. Formic and acetic acid are byproducts of hydrolysis, majorly they are byproducts of the breakdown of furans and phenolics, which are produced due to the degradation of polysaccharides and lignin. The integrated pretreatment techniques that are discussed in this work are illustrated in Fig. 6 with the yield of bioethanol produced in g/l from various feedstock used as the LCB.

## Cost-effective analysis of pretreatment strategies

The pretreatment of various feedstock was primarily responsible for the variations in energy consumption among various processes, with biomass processing being one of the biggest energy users and producers of greywater footprint. Despite these worries, it has been established that bioethanol made from diverse biomass has a lower environmental effect than gasoline alone. To evaluate the application of pretreatment at the pilot scale level, energy consumption throughout the pretreatment processes must be optimized [142]. Similarly, anaerobic biodegradation benefits from pretreatment techniques that have a constant energy balance to be a feasible process. For selecting an appropriate approach, the technological implementation of pretreatment methods from a lab to a pilot scale is crucial. Only the effects of liquefaction, increased biodegradability and bioethanol generation have been identified in lab-scale experiments. To implement on a pilot scale, it is required that bioethanol production be economical, energy-intensive and a green process that must be further addressed in near future [143]. Thus, the development of technically advanced and ecologically responsible biorefinery concepts based on technologies to valorize all of the components of lignocellulosic biomass (LCB), including lignin, is essential for the future of biomass-based production of bioethanol [144].

The development of the bioethanol process has made it among the sustainable bio-economies. For making the process sustainable, there is a need for adoption of a multi-layered strategy that includes the use of cutting-edge bioprocess technologies, integrated enzyme production technology, lower-cost raw materials as a source for enzyme synthesis as well as strain enhancement through mutation/bioengineering in biological pretreatment [142, 145]. The cost analysis of pretreatment processes covers 18% of the entire project cost for the manufacturing of cellulosic ethanol. As a pre-processing step, the cost of physical pretreatment was equal to 2% to 4% of the retrieved energy through the production of ethanol. The milling process used substantially more energy per kg (4.0–12.5 MJ/kg), but the steam explosion treatment required much less energy, i.e., up to 0.2–0.6 MJ/kg. In the aforementioned circumstances, the energy needed to produce milled biomass with 0.5 mm particle size was almost 22 times greater than the energy needed to treat with a steam explosion at 2.8 Mpa of pressure applied [146]. Thus, the synthesis of bioethanol from biomass resulted in lower operational and raw material costs. Low-cost feedstock, as well as less usage of chemicals, aid in the development of low-cost bioethanol production. Utilizing low-toxic chemicals and an innovative integrated process lowers the overall cost of production and has a lesser negative impact on the environment. As a result, there is a requirement for integrated techniques of pre-processing as an alternate platform technology for the Biomass Pre-processing Unit to further increase the overall economic efficiency of the biorefinery in light of the high transportation costs of feedstock with low bulk density.

In addition, the difficulties in implementing the suggested distinct pretreatment on a pilot level, as well as the energy balance research and cost analysis should be studied in advance before implementation in a particular field. It has also been evaluated that the problems that have not been resolved include the production of inhibitors, decreased hydrolysis efficiency, high waste disposal costs, poorer fermentation efficiency, insufficient lignin and cellulose separation, intense energy requirements, and outstanding chemical usage could also be addressed in much greater details.

## Challenges and future perspectives in LCB pretreatment

Pretreatment (which includes extrusion, a steam explosion associated with mild acids, a steam explosion mixed with mild thermo-alkali, ionic liquid, and surfactants associated with a steam explosion) was used to boost the conversion of sugar as well as the production of bioethanol. The application is the only factor considered when choosing a pretreatment method. Integrated methods that combine two or more pretreatment techniques are advantageous over the traditional distinct pretreatment process in which there is a reduction in the number of operating stages as well as a lowering in the production of undesired inhibitors [115]. To develop novel and more effective pretreatment methods for lignocellulosic feedstocks, which have the potential to provide positive outcomes, a substantial study is still needed. There are several difficulties involved in converting lignocellulosic biomass, from gathering raw materials to distillation. Pretreatment is one of the unit activities that use the greatest energy. It should be done while considering the minimal development of inhibitors, power needs, and effluent generation. A major obstacle to effective lignocellulosic biorefinery is the intrinsic resistance of lignocellulosic biomass, which is caused by its complicated structure and the inclusion of inhibitory elements, especially lignin. For lignocellulosic biocomponents to be fragmented, cellulose fibers' surface area and solubility need to be increased, and lignin to be removed or extracted. Effective biomass pretreatment procedures are required for biomass conversion to biofuel [84]. Numerous drawbacks of traditional pretreatment techniques include corrosion of equipment, high cost of maintenance, and the production of harmful byproducts and effluents. Recent years have seen the development of several single-step, multi-step, and/or combinations of physicochemical pretreatment methods that are easier to use, more cost-effective, and ecologically benign. Many of these coupled physicochemical techniques also increase biomass bio-accessibility and successfully separate 96% of LCB into cellulose, hemicellulose, and lignin, enabling extremely effective biomass processing [147]. But as we move towards diverse pretreatment methods for lignocellulosic biomasses (LCBs) now in use confront several difficulties, including the need for large amounts of energy and money as well as the potential for the formation of complicated inhibitors and environmental contamination. With the use of nanomaterials as nanocatalysts, these restrictions may be addressed during the preliminary step of the pretreatment of LCBs [148]. There is a greater level of applicability for nanomaterials in terms of their application towards organic understanding for biomass treatment, appropriateness of nanoparticles to microorganisms, and the opportunity to apply accessible innovation for efficient biofuel generation. As a result, nanomaterials biofuel production has enormous inherent potential, and much more research is required to solve the technological constraints on the manufacture of liquid biofuels [149]. Lignocellulosic materials from many sources could be used biochemically to make ethanol, however, large-scale production remains impractical due to poor yield, high water usage, and increased enzyme costs. Thus, developing novel pretreatment techniques, optimizing existing ones, and assessing the compatibility of new (typically residues) biomass sources are all current themes in the manufacture of 2G ethanol from LCB.

## Conclusion

Second-generation (lignocellulosic) bioethanol production appears to be the most promising renewable feedstock for meeting Sustainable Development Goals. Several feedstock pretreatments have revealed process challenges in terms of yield and product inhibitors. However, the pretreatment of lignocellulosic biomass is a crucial step towards bioethanol production from available biomass due to the recalcitrant structure of LCBs. It is required for the delignification of biomass, i.e., the removal of lignin to make the availability of cellulose and hemicellulose for further processes of saccharification. Till now, the known pretreatment methods, i.e., physical, chemical, biological, and physiochemical approaches are enacted. Further advancement in these processes is required to develop the combined pretreatment for economically feasible processes. The main focus is to develop an efficient pretreatment to remove the non-fermentable part of lignocellulosic biomass to get fermentable sugar. Subsequently, the combined process of pretreatment will lessen the incubation time for the process with more efficient desired outcomes. Thus, this will shorten the pretreatment time as well as it will help in developing various new combined pretreatment processes at the required temperature, pH and retention time. This review article is based on current and future aspects of various combined pretreatment processes performed on available feedstocks. This will help the researcher in further planning, selection and development of an effective pretreatment process that will disintegrate the recalcitrant structure of lignocellulosic biomass.

## Supplementary Information

**Additional file 1: Figure S1.** Illustrate mechanism of pretreatment techniques towards bioethanol production and its effect on feedstock with main emphasis on the size reduction, cellulose disruption along with hemicellulose and lignin depolarization [14, 18, 22]. **Figure S2.** Depicts diagrammatic representation showing effect of acid pretreatment on feedstock [55, 56]. **Figure S3.** Depicts the diagrammatical representation of combined pretreatment using chemical along with steam explosion process [32, 34].

## Data Availability

All data analysed are included within this article.

## Abbreviations

LCBs: Lignocellulosic biomass
MT: Million tonnes
GHG: Greenhouse gases
BMP: Ball milling pretreatment
FTIR: Fourier transform infrared spectroscopy
CrI: Crystallinity index
KU: KOH/urea
SCB: Sugarcane bagasse
PEG: Polyethylene glycerol
IL: Ionic liquid
AFEX: Ammonium fibre explosion
SAA: Soaking in aqueous ammonia
EA: Extractive ammonia
LHW: Liquid hot water
WAO: Wet air oxidation
SEKOH: Steam explosion assisted with potassium hydroxide
NaOH: Sodium hydroxide
H_2_SO_4_: Sulphuric acid
HCl: Hydrochloric acid
CO_2_: Carbon dioxide
HMF: Hydroxymethyl furfural
