# Supplementary material for: Strategies of pretreatment of feedstocks for optimized bioethanol production: distinct and integrated approaches
Source: Biotechnol Biofuels Bioprod. 2023 Mar 13;16:44. doi: 10.1186/s13068-023-02295-2 (PMC10012730; doi:10.1186/s13068-023-02295-2)
Supplement: Supplementary file 1 — Additional file 1: Figure S1. Illustrate mechanism of pretreatment techniques towards bioethanol production and its effect on feedstock with main emphasis on the size reduction, cellulose disruption along with hemicellulose and lignin depolarization [14, 18, 22]. Figure S2. Depicts diagrammatic representation showing effect of acid pretreatment on feedstock [55, 56]. Figure S3. Depicts the diagrammatical representation of combined pretreatment using chemical along with steam explosion process [32, 34]. [file 13068_2023_2295_MOESM1_ESM.docx]

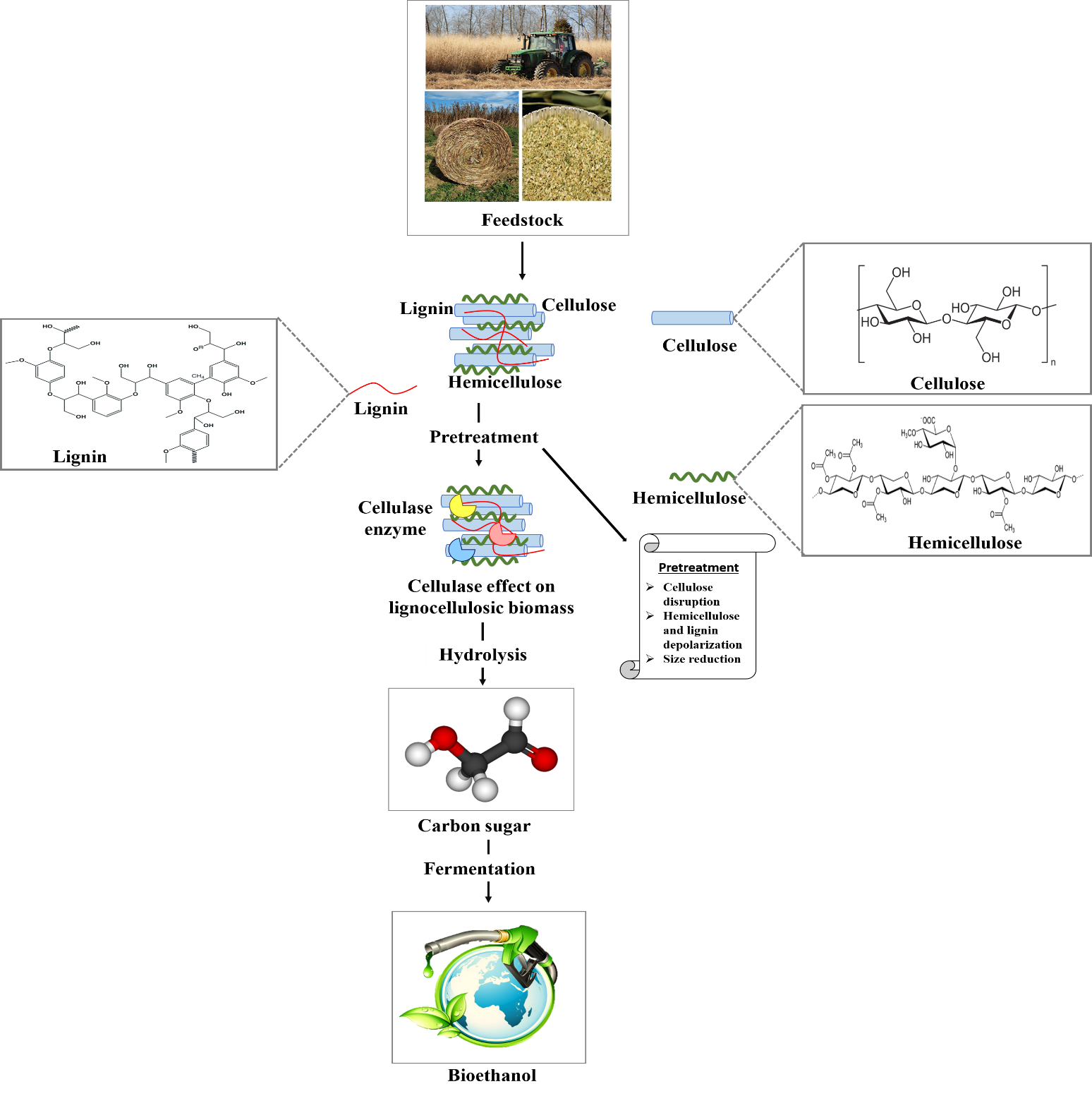


**Figure S1-** Illustrate mechanism of pretreatment techniques towards bioethanol production and its effect on feedstock with main emphasis on the size reduction, cellulose disruption along with hemicellulose and lignin depolarization [14,18,22].

**
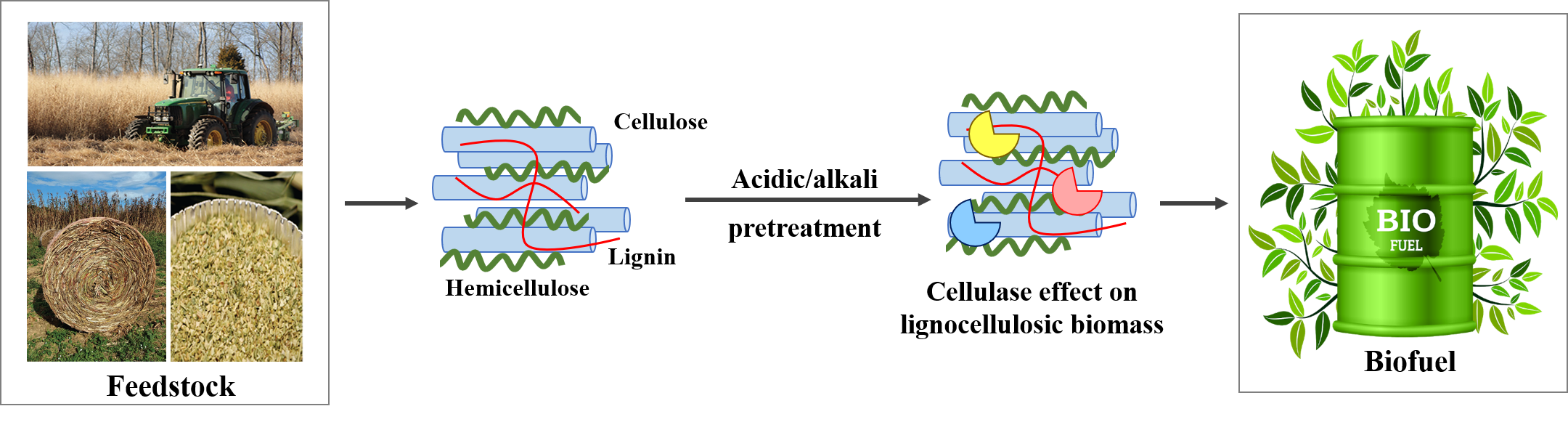
**

**Figure S2**- Depicts diagrammatic representation showing effect of acid pretreatment on feedstock [55,56].

**
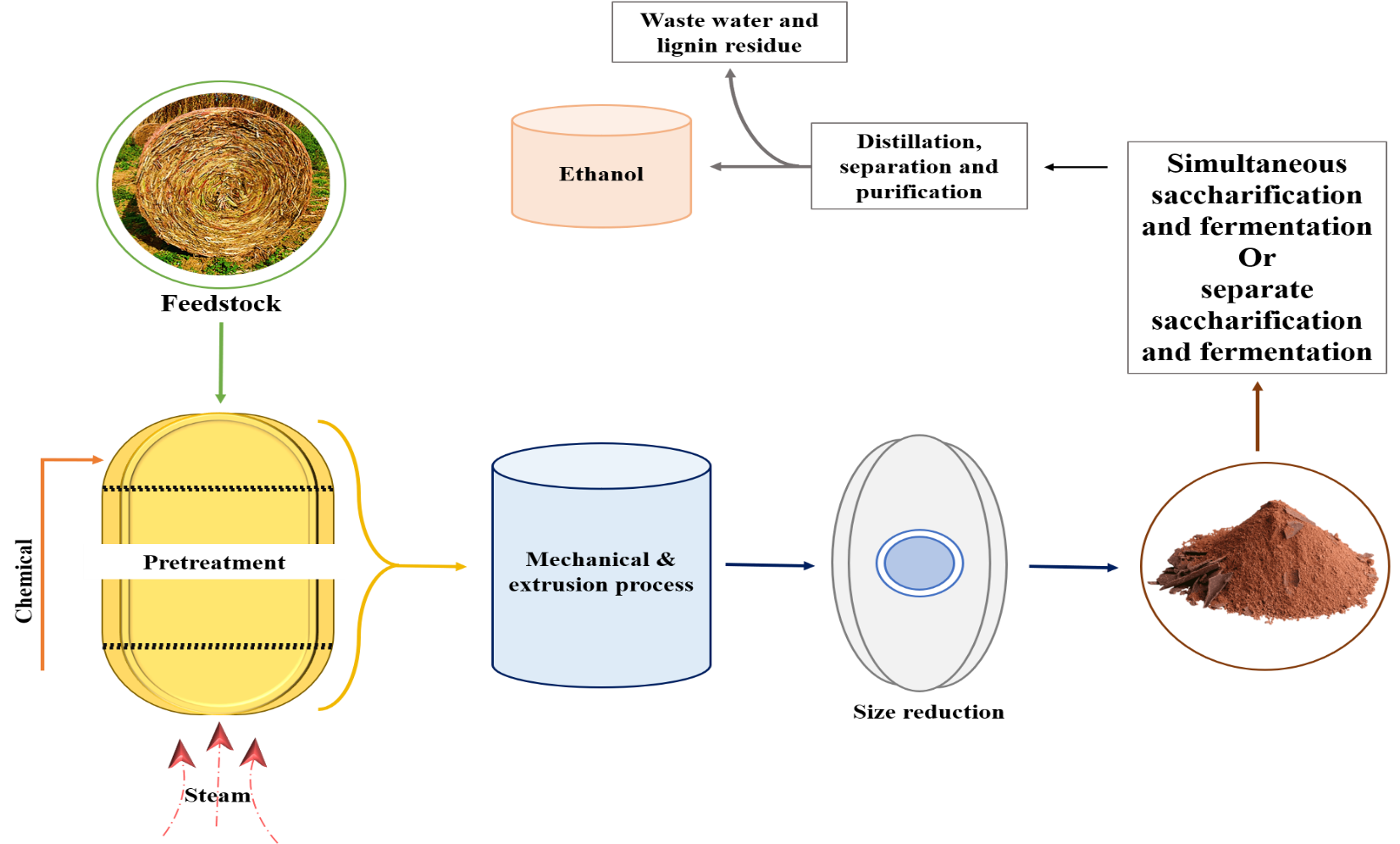
**

**Figure S3**- Depicts the diagrammatical representation of combined pretreatment using chemical along with steam explosion process [32,34].
